# Spiritual needs in Denmark: a population-based cross-sectional survey linked to Danish national registers

**DOI:** 10.1016/j.lanepe.2023.100602

**Published:** 2023-03-12

**Authors:** Tobias Anker Stripp, Sonja Wehberg, Arndt Büssing, Harold G. Koenig, Tracy A. Balboni, Tyler J. VanderWeele, Jens Søndergaard, Niels Christian Hvidt

**Affiliations:** a Research Unit for General Practice, Department of Public Health, University of Southern Denmark, Odense, Denmark; b Department of Epidemiology, Harvard T.H. Chan School of Public Health, Cambridge, MA, USA; c Human Flourishing Program, Institute for Quantitative Social Science, Harvard University, Cambridge, MA, USA; d Professorship Quality of Life, Spirituality and Coping, Faculty of Health, Witten/Herdecke University, Herdecke, Germany; e Department of Psychiatry and Behavioral Sciences, Duke University Medical Center, Durham, NC, USA; f Department of Medicine, Duke University Medical Center, Durham, NC, USA; g Division of Psychiatry, Department of Medicine, King Abdulaziz University, Jeddah, Saudi Arabia; h Dana-Farber/Brigham and Women's Cancer Center, Boston, MA, USA; i Harvard Medical School, Boston, MA, USA; j Academy for Geriatric Cancer Research (AgeCare), Odense University Hospital, Odense, Denmark

**Keywords:** Spiritual need, Spiritual care, Spirituality, Secular, Post-secular, Public health, Holistic care

## Abstract

**Background:**

Spiritual aspects of the human condition may give rise to spiritual pain and suffering, especially in the face of illness or difficult life situations. A growing volume of research documents the effects of religiosity, spirituality, meaning, and purpose on health. In supposedly secular societies, however, spiritual matters are rarely addressed in healthcare. This is the first large scale study to examine spiritual needs in Danish culture, and the largest study on spiritual needs to date.

**Methods:**

A population-based sample of 104,137 adult (≥18 yrs) Danes were surveyed cross-sectionally (the EXICODE study) and responses were linked to data from Danish national registers. The primary outcome was spiritual needs in four dimensions: religious, existential, generativity, and inner peace. Logistic regression models were fitted to examine the relationship between participant characteristics and spiritual needs.

**Findings:**

A total of 26,678 participants responded to the survey (25.6%). Of included participants 19,507 (81.9%) reported at least one strong or very strong spiritual need in the past month. The Danes scored highest on inner peace needs, followed by generativity, then existential, and lastly, religious needs. Affiliating as religious or spiritual, regularly meditating or praying, or reporting low health, low life satisfaction, or low well-being increased the odds of having spiritual needs.

**Interpretation:**

This study demonstrated that spiritual needs are common among Danes. These findings have important implications for public health policies and clinical care. Care for the spiritual dimension of health is warranted as part of holistic, person-centered care in what we term ‘post-secular’ societies. Future research should inform how spiritual needs might be addressed in healthy and diseased populations in Denmark and other European countries and the clinical effectiveness of such interventions.

**Funding:**

The paper was supported by the 10.13039/100008363Danish Cancer Society (R247-A14755), The 10.13039/100010809Jascha Foundation (ID 3610), The Danish Lung Foundation, AgeCare, and the 10.13039/501100006356University of Southern Denmark.

**Research in context**

**Evidence before this study**

While there is much literature on spiritual care and the positive effect of religion and spirituality on many health outcomes, scientific studies of how populations in supposedly secular societies experience spiritual needs are virtually non-existing. Spiritual aspects of the human condition can give rise to spiritual needs that may accentuate in the face of illness or difficult life situations. Despite decade-old recommendations, spiritual matters are rarely addressed in healthcare in what we term ‘post-secular’ societies. Most evidence for spiritual needs in Danes, who should be considered post-secular rather than secular, has been provided qualitatively. Thus, it is still unclear if spiritual needs are quantitatively present in Danes. Such evidence would have important implications for public health policies and clinical care and support the efforts and recommendations to include care for spiritual matters as part of holistic, patient-centered care in Danish society. PubMed was searched from inception until the 6th of February 2023 with the term: (“secular” OR “post-secular” OR “post secular” OR “postsecular”) AND (“spiritual needs” OR “spiritual need” OR “spiritual concern”), which yielded 27 hits. Since only quantitative evidence from randomly selected populations was considered, 0 studies met inclusion. The available evidence on spiritual needs is predominantly from relatively small European samples in different diseased populations, which were not possible to search up or compare systematically. Much evidence is available on spiritual well-being, but the clinically important measure of spiritual needs is gravely understudied in the Danish (and other) healthcare systems, where a patient-centered and holistic approach that includes care for spiritual matters is recommended.

**Added value of this study**

This study is the largest to examine spiritual needs to date. We show quantitatively for the first time that Danes, in what we term a ‘post-secular’ society, do indeed report spiritual needs. Further, we report specific individual characteristics that are associated with having different types of spiritual needs. Finally, we discuss how our findings are essential for public health policies and clinical care.

**Implications of all the available evidence**

Since it is evident that spirituality is essential for health, and persons from Denmark report spiritual needs, it is implied that care for such needs should be undertaken in Danish society. Since this is the case in Denmark, it might be similar in other post-secular European cultures. More research on how spiritual needs might best be addressed and the clinical effectiveness of such spiritual care interventions is warranted.

## Introduction

Although research supports the importance of spiritual matters for health, attention to spiritual care is generally uncommon in healthcare, especially in supposedly secular cultures. This is partly due to limited information on whether adults in secular cultures have spiritual needs and, if so, what kinds of spiritual needs. The present study examines whether adult Danes have spiritual needs, a finding that could have important implications for policymakers and healthcare professionals. Overall, we hypothesise that Danes, who should be seen as post-secular rather than merely secular, have concerns related to the spiritual dimensions of life and that these spiritual needs differ across the life span and sociodemographic, mental, and physical health status.

While persons are physical, psychological, and social beings, there is also a spiritual aspect of the human experience. Humans have striven to understand the purpose and meaning of life since the earliest times and continue to do so.^1^ The histories of religion and philosophy are replete with such reflection. All persons experience this search for meaning and purpose to some extent, and consequently is also an essential part of healthcare. For this study, we use the European version of the U.S. consensus definition^2^ of ‘spirituality’: “Spirituality is the dynamic dimension of human life that relates to the way persons (individual and community) experience, express and/or seek meaning, purpose and transcendence, and the way they connect to the moment, to self, to others, to nature, to the significant and/or the sacred.^3^” This definition is inclusive of theistic (e.g., religious), nontheistic, and atheistic (e.g., classic secular existential) aspects of the human condition. ‘Spiritual needs’ are thus considered needs, concerns, or suffering related to the above understanding of spirituality. The care administered to address ‘spiritual needs’ is called ‘spiritual care’.^4^ Spiritual care not only addresses suffering and distress but also seeks to support patients' spiritual resources and practices. The academic discussion on religious, spiritual, and existential definitions and their relation to (or overlap with) mental processes is complex.^5^, ^6^, ^7^, ^8^ However, expanding this discussion is not our manuscript's primary purpose, and readers interested in this topic are encouraged to look elsewhere. We consider the spiritual dimension to be a distinct part of health, along with the physical, mental, and social.^9^^,^^10^

Several reasons exist why spiritual needs are essential to healthcare. First, research increasingly demonstrates that spirituality and religiosity may have protective effects on health.^11^, ^12^, ^13^, ^14^ Second, patients confronted with a severe illness often experience spiritual needs such as a crisis of meaning, loss of hope, or fear of death—and may need help dealing with such considerations.^15^^,^^16^ Third, providing spiritual care could have important implications for health care professionals,^17^ patient health outcomes,^18^^,^^19^ and costs of care.^20^ A recent comprehensive review emphasised the effects of spirituality on health and provided recommendations on how to approach spiritual issues in healthcare.^14^ Fourth, although the World Health Organization (WHO) has grappled with how to understand a spiritual dimension of health,^9^ they have developed various tools to try to measure such a dimension (e.g., the WHOQOL-SRPB^21^) underlining its importance. In addition, the WHO has (in defining palliative care), along with the World Organizations of Family Doctors (in defining primary care), and many other professional groups, incorporated spiritual issues as important aspects of healthcare over the past decades. The Lancet Commission on Palliative Care similarly considered spiritual suffering and pain on the same level of importance as physical and psychosocial pain.^10^ While there may be a bias in the research field towards conclusions that endorse a greater role of religion in healthcare with well-known voices criticising such bias^22^ there is broad consensus that the relationship between religion in health is substantial and should be part of patient-centered healthcare.^14^

In contemporary Denmark, the religious and spiritual landscape is complex. Approximately 75% of Danes are paying members of the Evangelical-Lutheran Church, while a mere 2% attend religious service weekly. Faith and belief are societal taboos, second only to mental health disorders in magnitude.^23^ Spirituality, however, is very much present and, according to some data, is growing in the population, but is, it seems, practised and dealt with privately. Following the notion introduced by Habermas, we thus consider Denmark a ‘post-secular’ culture.^24^ By this, we acknowledge that the traditionally secular and non-seculawr spheres are constantly mixed at macro and micro levels, that spirituality is important and present in the society and in Danes, and that religion and spirituality have not declined as expected with the increase of “knowledge”.^25^

The spiritual dimension of health can be measured by various questionnaires, e.g., the WHOQOL-SRPB or the SWBS,^26^ to name a few of the most widely used. For this study, we were interested in a tool that, being more directly clinically transferrable, measured ‘spiritual needs’.^27^ One of the most widely used instruments to do this is the Spiritual Needs Questionnaire (SpNQ).^28^ The SpNQ (20 items), which has been psychometrically validated in a variety of populations and languages, measures spiritual needs in four main dimensions originally identified through exploratory factor analysis (see Box 1): (1) ‘religious needs,’ which cover needs related to the transcendent, e.g., God, Allah, the Universe, or other higher power, as well as needs related to religious communities and practices; (2) ‘existential needs,’ which cover those related to reflections on life and death, meaning in life, etc.; (3) ‘generativity needs,’ which relate to the need to give something to others and help other people; and lastly, (4) ‘inner peace needs,’ which cover needs related to a sense of inner peace or rest in nature.^30^ Importantly, the SpNQ is not limited to a particular subgroup of believers that adhere to a specific religious or spiritual community and has been used in various countries with diverse cultural and religious backgrounds.^31^ For some items, examples of specific observances are given to increase content validity and comprehensibility,^32^ but respondents are free to respond based on important aspects of their own experiences and life. A tool such as the SpNQ enables quantitative examination of spiritual needs in a post-secular context as it broadly addresses spiritual matters without focusing solely on e.g., traditional religious topics.

Box 1 **Conceptual content of the spiritual needs questionnaire.**

**Religious needs:**
  ○ Pray for yourself
  ○ Pray with someone
  ○ Someone prays for you
  ○ Turn to a higher presence e.g. God, Allah, the Universe
  ○ Participate in a religious ceremony (e.g. service)
  ○ Read religious/spiritual books
**Existential needs:**
  ○ Be forgiven
  ○ Forgive someone
  ○ Dissolve open aspects of your life
  ○ Talk about the question of meaning in life
  ○ Talk about the possibility of life after death
  ○ Find meaning in illness and/or suffering
**Inner peace needs:**
  ○ Dwell at a place of quietness and peace
  ○ Plunge into beauty of nature
  ○ Find inner peace
  ○ Talk with someone about fears and worries
**Generativity needs:**
  ○ Pass own life experience to others
  ○ Be assured that your life was meaningful and of value
  ○ Give solace to someone
  ○ Give away something from yourself

As many studies of spiritual needs have focused on religious cultural contexts, robust and high-quality examinations of spiritual needs in post-secular cultures are essential to guide spiritual care for these populations. Furthermore, this may help inform the administration of spiritual care by mapping which persons typically have which types of need, thus enabling more specific and person-centered care.^29^ Such examinations in the general population have the potential to inform important public policy decisions that support a holistic healthcare system where physical, mental, social, and spiritual aspects of health are recognised and addressed.

### Objectives

In the present cross-sectional study, we sought to measure spiritual needs in the adult Danish population by using survey and national register data collected in the EXIstential health COhort DEnmark (EXICODE) study.^33^ More specifically, we:

1) estimated the prevalence of spiritual needs in a large representative sample of adult Danes; and
2) examined associations between demographic characteristics and spiritual needs using multivariate logistic regression.

## Methods

This study is based on survey and national health register data collected in EXICODE. The design and methodology of the EXICODE study have been reported elsewhere,^33^ but are now summarised here with a focus on the objectives above. Information on instrument selection, translation, and the cognitive interviews performed to test the questionnaire is also reported elsewhere.^32^ EXICODE is based on a Danish national digital survey linked to individual-level data from comprehensive Danish nationwide health registers.^33^^,^^34^

### Study design, population and inclusion criteria

**Fig. 1:**
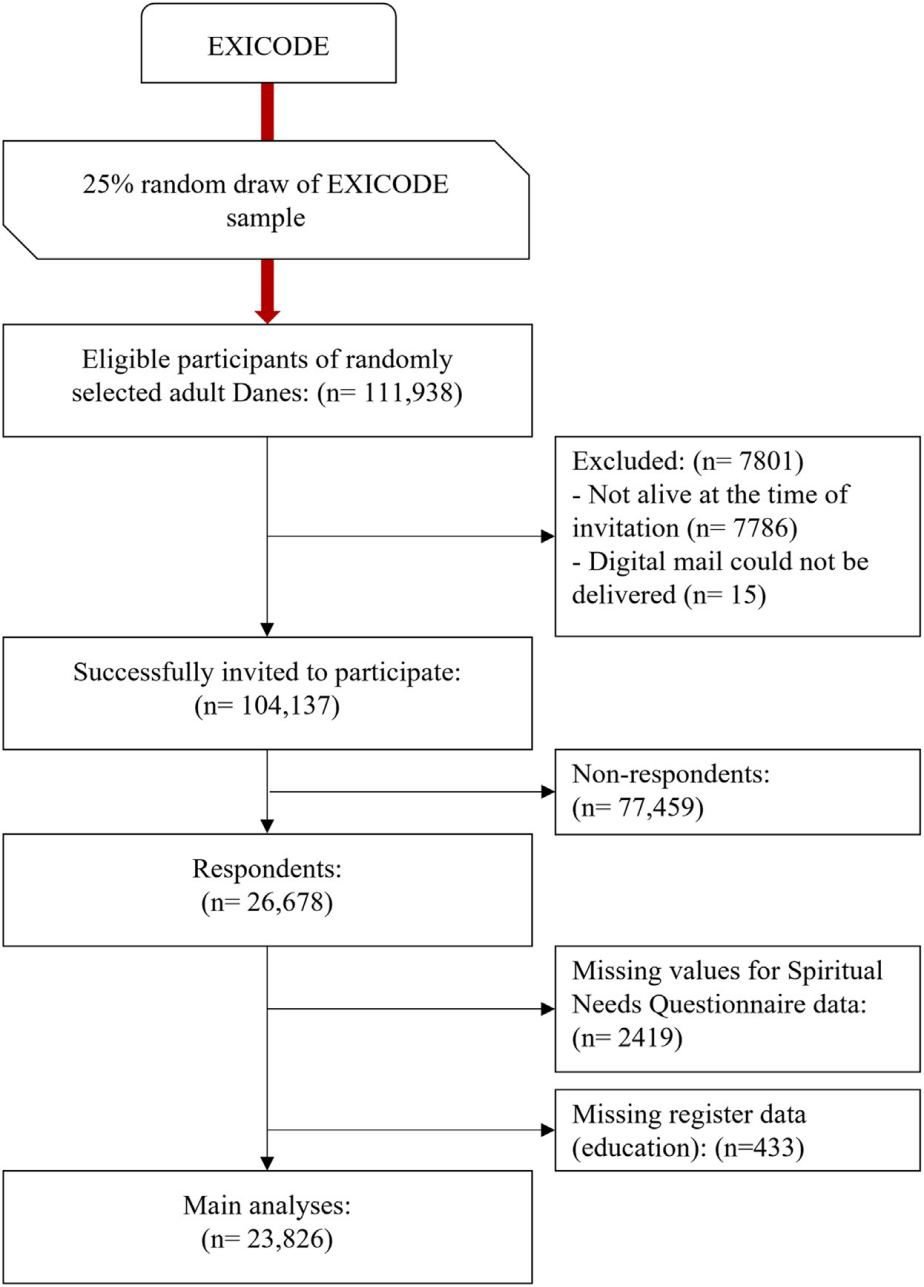
Flowchart of study sampling and participants.

Initially, a 10% random sample of the adult Danish population (≥18 yrs) was identified by the Danish Health Data Authority (SDS)^34^ through CPR-numbers (a unique 10-digit personal identifier code that all Danish citizens have). The primary inclusion criterion was age ≥18 yrs on January 1st, 2020. For the present study, 25% of that sample was randomly chosen to be invited to participate in the first wave of EXICODE (Fig. 1). The study was conducted between the 1st of November and the 13th of December 2021 (with a single reminder).

In total, 111,938 participants were eligible. Of those, 7801 were excluded since they were no longer alive when invited to participate or did not receive digital mail, leaving 104,137 adult Danes who were successfully invited to participate in Wave I of the EXICODE digital survey. Survey data were subsequently linked to Danish national registries.

### Ethics

The ethics statement is similar to the one reported in the EXICODE protocol paper.^33^ The project was registered for legal and GDPR concerns at the *University of Southern Denmark Legal Services* (SDU RIO) (journal number: 10.367). Two institutions declared that the project did not require ethical approval by Danish law: the *Danish Regional Scientific Ethical Committee* (journal number: 20202000-116) and the *Danish Authority for Patient Security* (STPS). However, due to institutional best practices, the project was evaluated and approved by the institutional ethics review board *the University of Southern Denmark Research Ethics Committee* (SDU REC) (journal number: 20/39546). The project follows *The Danish Code of Conduct for Research Integrity* and is carried out following the *Helsinki Declaration*.

### Measures

#### Register variables

Age and sex were determined from the CPR-number. Age was categorised in groups by years as 18–25, 26–35, 36–45, 46–55, 56–65, 66–75, and 75+. Sex was considered binary as either male (0) or female (1). Demographic register data were drawn from 2019 to obtain pre-pandemic demographic information. Education was measured in years based on the ISCED-system and categorised as 7–11, 12–14, and 15+ years. Income was based on household income and operationalised in tertiles relative to age in decile groups as low, middle, and high, i.e., allowing for income groups relative to people of similar age. For regression analyses, income and education categories were combined to yield three levels of socioeconomic status (SES): low (low income + 7–11 or 12–14 years of education and middle income + 7–11 years of education), medium (low income + 15+ years education, middle income + 12–14 years education, and high income + 7–11 years education), and high (medium income + 15+ years education and high income + 12–14 or 15+ years education). Cohabitation was categorised as living with someone (partner) versus living alone. Work status was categorised as either working or being on benefit (student, public, or pension). Civil status was classified as married, widowed, divorced, or unmarried. Chronic diseases were operationalised as having none, one, or more than one of eight common chronic diseases (asthma, dementia, chronic obstructive pulmonary disease (COPD), arthritis, osteoporosis, schizophrenia, type 1-diabetes, type 2-diabetes).

### Outcomes

#### Primary outcome

The primary outcome of this study was the intensity of spiritual needs. These were measured using the Danish 20-item Spiritual Needs Questionnaire (DA-SpNQ-20) and categorised as religious (6 items), existential (6 items), generativity (4 items), and inner peace (4 items) needs.^30^ The instrument was tested in a convenience sample of relatively healthy, young, primarily female adults, and demonstrated satisfactory content and structural validity. Internal reliability was considered adequate across dimensions (Cronbach's α = 0.73–0.93), as was the test-retest reliability (ICC2.1 = 0.86). Items were scored on a 4-point scale from no need to very strong need (0—no, 1—somewhat, 2—strong, 3—very strong). The scale may be considered ordinal and was transformed to a linear score by computing mean scores for each dimension. An overall mean score for the 20-item SpNQ was also calculated. For logistic regression analyses the mean scores overall and per dimension were dichotomised as mean score ≥0.5 (equivalent to scoring at least “1—Somewhat” in 10 items, or “2—Strong” in 5 items) = “having spirituals needs” and mean score <0.5 = “not having spiritual needs”.

#### Secondary outcomes

Self-perceived physical health was derived from the D2.1 item of the Flourish Index^35^ as “In general, how would you rate your physical health?” and scored on an 11-point VAS-scale from 0 = poor to 10 = excellent.

The WHO-5 (World Health Organization Well-Being Index) is a 5-item scale measuring subjective mental well-being.^36^ It has acceptable construct validity and responsiveness. It is a unidimensional instrument (e.g., Cronbach's α = 0.82) that has been validated and tested in multiple populations worldwide. Items are scored on a 6-point scale from ‘0—never’ to ‘5—all the time’. Summed scores are transformed into a scale ranging from 0 to 100 by multiplying by 4.

The following items measured meaning in life and crisis of meaning: “I see a meaning in my life”; “My life is meaningful”; “I experience that my life is absolutely worth living”; “I suffer from not being able to see a meaning in my life”; “I lack a meaning in my life”; and “My life seems empty”. Some of these items are from Tatjana Schnell's Sources of Meaning Questionnaire.^37^ Items are scored on a 6-point Likert scale from ‘0—don't agree’ to ‘5—totally agree’, and the score for meaning and crisis of meaning is the mean score of the three first and last items, respectively.

The Brief Multidimensional Life Satisfaction Scale (BMLSS-10) is a 10-item instrument that quantifies satisfaction with five main aspects of life, i.e., intrinsic, social, external, perspective, and health.^38^ It has been tested in healthy elderly and in patients with chronic diseases. It has a single-factor structure (Cronbach's α = 0.92). The BMLSS is scored on a 7-point Likert scale from ‘0—very unsatisfied’ to ‘7—very satisfied’. The score is transformed into a scale ranging from 0 to 100.

Single items were used for spiritual beliefs and practices. The items were: “Do you consider yourself a believer, non-believer, convinced atheist, or don't know?”; “If believer, what faith?”; “Do you consider yourself religious, spiritual, religious and spiritual, or none?”; and “Do you believe in an afterlife?” Included here was a VAS scale from 1 to 10 for assessing “How big a role does God play in your life today?” and “How big a role did God play in your life when you were a child/young?” Finally, we measured specific spiritual practices with the items: “How often do you (1) pray privately (for yourself or others)?; (2) meditate (any form)?; and (3) go to church?” Response options were dichotomised into “regularly”/“often” as *Yes* and “seldom”/“never” as *No*.

### Statistical analyses

Descriptive statistics (frequencies and percentages) of the sample (by sex and respondents/non-respondents) and the prevalence of spiritual beliefs and practices (by sex) were calculated. Differences were tested by chi-squared tests.

Multiple univariate and multivariate logistic regression models were fitted to estimate the odds ratio (OR) for having spiritual needs by demographic characteristics (age, sex, SES, habitation, civil status, and chronic diseases) and health characteristics (self-perceived physical health, well-being, satisfaction with life, meaning in life, crisis of meaning), as well as for various spiritual beliefs and practices. Model 1: Demographic variables were fitted in multivariable models on SpNQ dimensions. Model 2: Both demographic and surveyed variables on spiritual beliefs and practices were fitted in large multivariable models to examine associations with spiritual needs dimensions. For categorical variables, we performed Wald tests for overall statistical significance in the multivariable models. Linear regression models for continuous outcomes were fitted and are reported in the appendix. In addition, we computed corresponding regression models for each of the four-dimensional scores of spiritual needs (religious, existential, generativity, and inner peace) separately.

The online survey system forced responses to all questions on a page before it could continue to the next set of questions. Thus, missing items occurred only when (partial) responders discontinued the survey; all variables following their last entry would be “missing values”. Since partial responders have dropped out at various points in the (rather long) survey, we consider the missing values to be missing at random. Since the SpNQ was the primary outcome, partial responses for this variable were excluded (Fig. 1). Missing values for categorical variables (2–4%) were assigned to the majority category (which only altered the interpretation). Missing values for continuous variables (0–2%) were set to the overall mean level.

All statistical analyses were performed using STATA 17, and the alpha level for statistical significance was set at 0.05.

### Role of the funding source

None of the funders had any influence in the design, conduction, analysis, writing of or decision to submit this research.

## Results

### Sample

In total, 26,678 of the selected Danes responded, yielding a response rate of 25.6%. Respondent analyses were conducted to examine differences in demographic variables between respondents and non-respondents (see Appendix 1 for respondent table). Respondents differed from non-respondents on all measured demographic variables. Respondents were more likely to be female, older, better educated, had higher income, and were more inclined to live with someone, be married, and be retired/living on a pension than non-respondents. Among respondents, 23,826 participants had complete SpNQ and register datasets (Table 1), of which 55.7% were female, and the mean age was 55.66 years (SD = 16.27; range = 18–98).

**Table 1:** Demographic variables of the sample of randomly selected adult Danes included in analyses by sex (n = 23,826).

	All	Female	Male
**Total**	23,826 (100.0)	13,277 (100.0)	10,549 (100.0)
**Age, years**			
18–25	1360 (5.7)	838 (6.3)	522 (4.9)
26–35	2042 (8.6)	1295 (9.8)	747 (7.1)
36–45	2643 (11.1)	1648 (12.4)	995 (9.4)
46–55	4610 (19.3)	2679 (20.2)	1931 (18.3)
56–65	5598 (23.5)	3057 (23.0)	2541 (24.1)
66–75	5390 (22.6)	2733 (20.6)	2657 (25.2)
75+	2183 (9.2)	1027 (7.7)	1156 (11.0)
**Education, years**			
7−	4051 (17.0)	2246 (16.9)	1805 (17.1)
12−	10,031 (42.1)	5359 (40.4)	4672 (44.3)
15−	9744 (40.9)	5672 (42.7)	4072 (38.6)
**Income tertiles, relative to age**			
Lower	5485 (23.0)	3343 (25.2)	2142 (20.3)
Middle	8347 (35.0)	4700 (35.4)	3647 (34.6)
Upper	9994 (41.9)	5234 (39.4)	4760 (45.1)
**Living status**			
Living alone	6846 (28.7)	4162 (31.3)	2684 (25.4)
Living with someone	16,980 (71.3)	9115 (68.7)	7865 (74.6)
**Working status**			
Working	11,087 (46.5)	5974 (45.0)	5113 (48.5)
Student benefit	863 (3.6)	586 (4.4)	277 (2.6)
Other public benefit	4898 (20.6)	3254 (24.5)	1644 (15.6)
Public and private pension(s)	6978 (29.3)	3463 (26.1)	3515 (33.3)
**Civil status**			
Married	13,960 (58.6)	7372 (55.5)	6588 (62.5)
Widow/er	1293 (5.4)	917 (6.9)	376 (3.6)
Divorced	2969 (12.5)	1817 (13.7)	1152 (10.9)
Unmarried	5604 (23.5)	3171 (23.9)	2433 (23.1)
**Chronic disease**			
No	18,557 (77.9)	10,134 (76.3)	8423 (79.8)
Yes, one	4684 (19.7)	2736 (20.6)	1948 (18.5)
More than one	585 (2.5)	407 (3.1)	178 (1.7)

All groups were statistically significantly different at p < 0.01, based on chi-squared tests.

More female respondents were believers and/or religious, spiritual, religious and spiritual compared to male respondents who more frequently considered themselves non-believers or convinced atheists and neither religious nor spiritual or none of them (Table 2). As expected, and in accord with the Danish national church being Christian Protestant, a large portion of respondents (30.9%) who indicated themselves to be believers were affiliated with Protestant Christianity. However, agnostics, Buddhists, Hindus, Muslims, Jews, Catholics, Aesir-faith believers (Aesir faith is the traditional old/pre-Christian faith in the Nordic region, which is still practised as a minority religion today), and universal faith w/o specific theology believers, and others, were also present in the sample. Of respondents, 41.5% did not believe in life after death, with 20.4% and 27.1% respectively either believing in life after death or didn't know what they thought of it, respectively. Furthermore, most Danes did not consider God to play an important role in their lives today (75.6%) and did not consider God to have played an important role when they were a child/young (68.9%). There were, however, sex differences, with women being more inclined to indicate that God was important to them (both today and when a child/young). The same was true for spiritual practices: women were more inclined than men to indicate that they pray (34% vs. 16%), meditate (27% vs. 13%), or visit church (17% vs. 11%).

**Table 2:** Spiritual beliefs and practices (n = 23,826).

	All	Female	Male
	23,826 (100.0)	13,277 (100.0)	10,549 (100.0)
**Do you consider yourself as…**			
Believer	9869 (41.4)	6007 (45.2)	3862 (36.6)
Non-believer	6415 (26.9)	3095 (23.3)	3320 (31.5)
Convinced atheist	2161 (9.1)	855 (6.4)	1306 (12.4)
Don't know	5381 (22.6)	3320 (25.0)	2061 (19.5)
**If believer, then what faith?**			
Agnosticism	414 (1.7)	173 (1.3)	241 (2.3)
Buddhism	229 (1.0)	146 (1.1)	83 (0.8)
Hinduism	29 (0.1)	15 (0.1)	14 (0.1)
Islam	179 (0.8)	110 (0.8)	69 (0.7)
Judaism	16 (0.1)	7 (0.1)	9 (0.1)
Christianity: catholicism	534 (2.2)	310 (2.3)	224 (2.1)
Christianity: protestantism	8059 (33.8)	4752 (35.8)	3307 (31.3)
Aesir faith^a^	94 (0.4)	26 (0.2)	68 (0.6)
Universal faith w/o specific theology	911 (3.8)	646 (4.9)	265 (2.5)
Other	384 (1.6)	229 (1.7)	155 (1.5)
Don't know	752 (3.2)	444 (3.3)	308 (2.9)
Not answered/missing	12,225 (51.3)	6419 (48.3)	5806 (55.0)
**What denomination suits you best?**			
None/don't know/missing	16,272 (68.3)	8372 (63.1)	7900 (74.9)
Religious	4095 (17.2)	2380 (17.9)	1715 (16.3)
Spiritual	1959 (8.2)	1417 (10.7)	542 (5.1)
Religious and spiritual	1500 (6.3)	1108 (8.3)	392 (3.7)
**Do you believe in an afterlife?**			
No/missing	11,381 (47.8)	5033 (37.9)	6348 (60.2)
Yes	5320 (22.3)	3699 (27.9)	1621 (15.4)
Don't know	7125 (29.9)	4545 (34.2)	2580 (24.5)
**How important is god in your life today?**			
Not important/missing	19,158 (80.4)	10,326 (77.8)	8832 (83.7)
Important	4668 (19.6)	2951 (22.2)	1717 (16.3)
**How important was god in your life as a child?**			
Not important/missing	18,172 (76.3)	9839 (74.1)	8333 (79.0)
Important	5654 (23.7)	3438 (25.9)	2216 (21.0)
**Do you pray?**			
No: rarely, never, missing	18,983 (79.7)	9908 (74.6)	9075 (86.0)
Yes: regularly, often	4843 (20.3)	3369 (25.4)	1474 (14.0)
**Do you meditate (any form)?**			
No: rarely, never, missing	19,740 (82.9)	10,431 (78.6)	9309 (88.2)
Yes: regularly, often	4086 (17.1)	2846 (21.4)	1240 (11.8)
**Do you go to church?**			
No: rarely, never, missing	20,887 (87.7)	11,350 (85.5)	9537 (90.4)
Yes: regularly, often	2939 (12.3)	1927 (14.5)	1012 (9.6)

All groups were statistically significantly different at p < 0.001, based on Chi-squared tests.

a Aesir faith is the traditional old/pre-Christian faith in the Nordic region which is still practiced as a minority religion today.

### Spiritual needs

For the full 20-item scale, 19,507 (81.9%) reported at least one strong or very strong (score of 2 or 3) spiritual need within the past month. Within specific SpNQ domains, 16,366 (68.7%) reported at least one inner peace need, 15,077 (63.3%) at least one generativity need, 9887 (41.5%) at least one existential need, and 4257 (17.9%) at least one religious need.

Danes in this sample were overall more likely to report having spiritual needs in the past 30 days than not (overall SpNQ mean score for the entire sample = 0.71, SD = 0.51), although variation across demographics was present (see Appendix 2 for distribution of spiritual needs by demographics). The prevalence of spiritual needs per dimension in ranked order were (1) Inner Peace needs (mean = 1.21), (2) Generativity needs (mean = 1.08), (3) Existential needs (mean = 0.54), and (4) Religious needs (mean = 0.29). Females generally reported a higher level of spiritual needs across all four domains. Respondents with ≥15 years of education likewise reported higher levels of spiritual needs across all domains (except for Existential needs). People in the lowest income tertile reported the highest levels of spiritual needs overall and across all dimensions compared to people in the middle or highest income tertile. Similarly, people who lived alone reported higher spiritual needs overall and across all dimensions compared to people who lived with someone.

### Correlates of spiritual needs

**Fig. 2:**
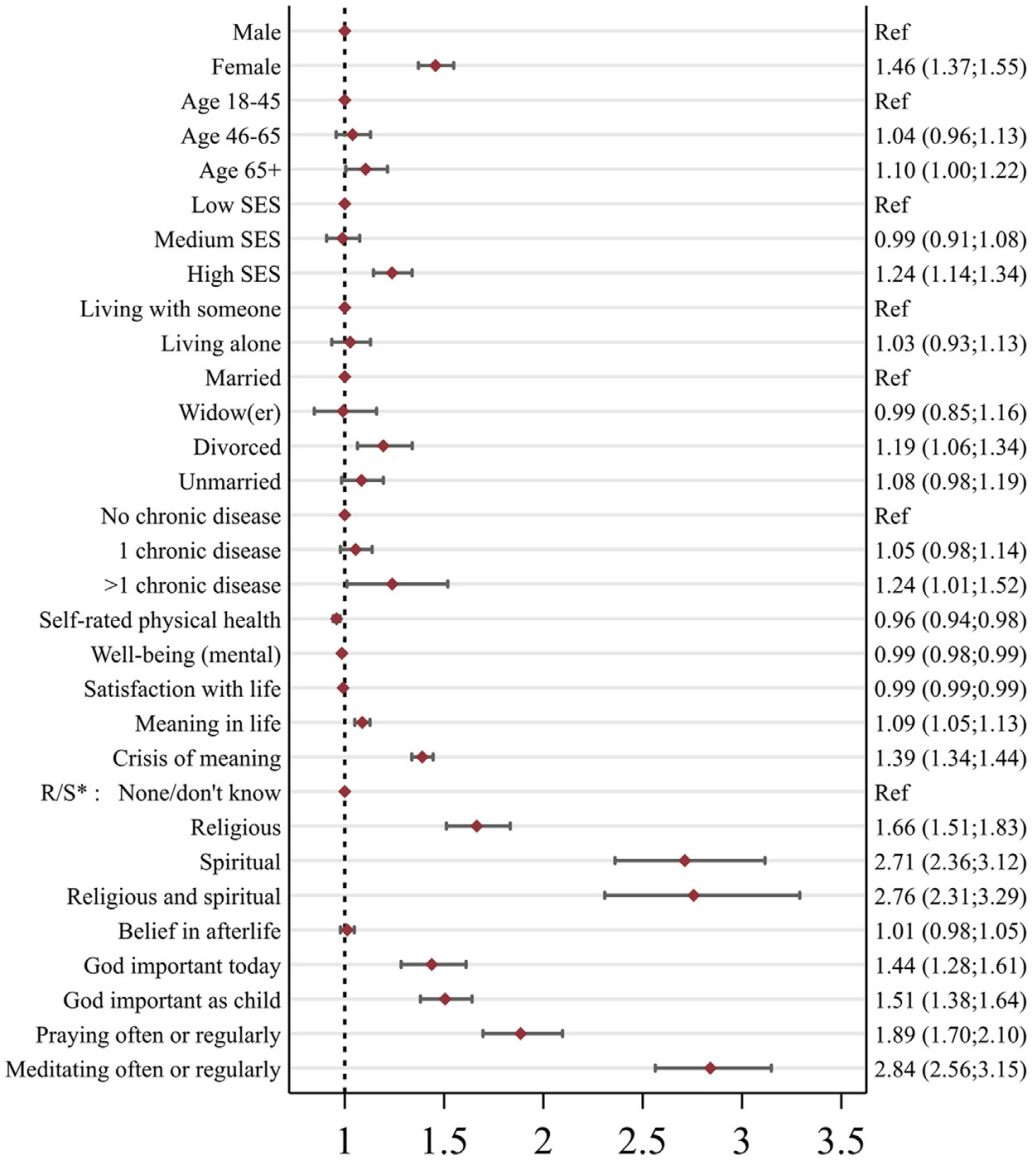
Forest plot of multivariable logistic regression model estimates showing odds ratio of spiritual needs and corresponding 95% confidence intervals by demographic variables and spiritual beliefs and practices (n = 23,826).

Results from the multivariable logistic regression models examining overall spiritual needs are presented in Table 3 and Fig. 2. The same analyses for each SpNQ dimension i.e. religious, existential, generativity, and inner peace needs are found in Appendices 3 and 4. Appendices 5 and 6 contain multivariable linear regressions by spiritual needs overall and dimensions.

**Table 3:** Uni- and multivariable logistic regression model estimates (odds ratios) of associations between spiritual needs (overall) and demographic and spiritual beliefs and practices (n = 23,826).

Variables	Univariable models		Model 1 (pseudo r^2^ = 0.02)		Model 2 (pseudo r^2^ = 0.16)	
	Odds ratio	p-val	Odds ratio	p-val	Odds ratio	p-val
Constant			1.36 (1.20; 1.55)	<0.001	2.22 (1.71; 2.87)	<0.001
Male	Ref	Ref	Ref		Ref	
Female	1.80 (1.71; 1.90)	<0.001	1.76 (1.67; 1.86)	<0.001	1.46 (1.37; 1.55)	<0.001
Age 18–45	Ref	Ref	Ref		Ref	0.1^a^
Age 46–65	0.86 (0.81; 0.92)	<0.001	0.91 (0.85; 0.98)	0.02	1.04 (0.96; 1.13)	0.36
Age 65+	0.84 (0.78; 0.90)	<0.001	0.93 (0.85; 1.01)	0.07	1.11 (1.01; 1.22)	0.04
Low ses	Ref	Ref	Ref	0.002^a^	Ref	<0.001^a^
Medium ses	0.85 (0.79; 0.91)	<0.001	0.92 (0.85; 0.99)	0.03	0.99 (0.91; 1.08)	0.78
High ses	0.91 (0.85; 0.97)	0.004	1.03 (0.96; 1.10)	0.47	1.24 (1.15; 1.34)	<0.001
Living alone	Ref	Ref	Ref		Ref	
Living with someone	0.74 (0.70; 0.79)	<0.001	0.82 (0.76; 0.90)	<0.001	1.03 (0.94; 1.13)	0.56
Married	Ref	Ref	Ref	<0.001^a^	Ref	0.01^a^
Widow(er)	1.17 (1.04; 1.32)	0.008	0.88 (0.76; 1.01)	0.07	0.99 (0.85; 1.16)	0.91
Divorced	1.44 (1.33; 1.57)	<0.001	1.21 (1.09; 1.34)	<0.001	1.19 (1.06; 1.34)	0.003
Unmarried	1.26 (1.18; 1.34)	<0.001	1.09 (1.00; 1.20)	0.04	1.08 (0.98; 1.19)	0.10
No chronic disease	Ref	Ref	Ref	<0.001^a^	Ref	0.06^a^
1 chronic disease	1.17 (1.11; 1.27)	<0.001	1.17 (1.09; 1.25)	<0.001	1.06 (0.98; 1.14)	0.17
>1 chronic disease	1.64 (1.36; 1.96)	<0.001	1.54 (1.28; 1.85)	<0.001	1.24 (1.01; 1.52)	0.04
Self-rated physical health	0.86 (0.85; 0.87)	<0.001			0.96 (0.94; 0.98)	<0.001
Well-being (mental)	0.98 (0.97; 0.98)	<0.001			0.99 (0.98; 0.99)	<0.001
Satisfaction with life	0.97 (0.97; 0.97)	<0.001			0.99 (0.99; 1.00)	<0.001
Meaning in life	0.81 (0.79; 0.83)	<0.001			1.09 (1.05; 1.13)	<0.001
Crisis of meaning	1.53 (1.49; 1.58)	<0.001			1.39 (1.34; 1.45)	<0.001
R/s∗: none/don't know	Ref	Ref			Ref	<0.001^a^
Religious	2.65 (2.45; 2.86)	<0.001			1.67 (1.51; 1.83)	<0.001
Spiritual	4.96 (4.36; 5.63)	<0.001			2.71 (2.36; 3.12)	<0.001
Religious and spiritual	6.72 (5.72; 7.90)	<0.001			2.76 (2.31; 3.29)	<0.001
Belief in afterlife	1.28 (1.24; 1.32)	<0.001			1.01 (0.98; 1.05)	0.48
God important today	3.62 (3.34; 3.92)	<0.001			1.44 (1.28; 1.61)	<0.001
God important as child	2.69 (2.51; 2.88)	<0.001			1.51 (1.38; 1.64)	<0.001
Praying often or regularly	4.33 (3.99; 4.71)	<0.001			1.89 (1.70; 2.10)	<0.001
Meditating often or regularly	4.81 (4.38; 5.28)	<0.001			2.84 (2.56; 3.15)	<0.001

Model 1 = spiritual needs as dependent variable; demographic variables as independent variables. Model 2 = spiritual needs as dependent variable; demographic variables and surveyed outcomes as independent variables.

a Wald test estimates for categorical variables.

#### Overall spiritual needs

Overall, being female, having high SES, being divorced, having meaning in life or a crisis of meaning (crisis 3-fold association size), being religious, spiritual or both, considering God important today and as child, as well as meditating or praying often all had positive ORs for having spiritual needs within the last 30 days. Self-rated health, well-being, and satisfaction with life were negatively associated with spiritual needs. The OR was strongest for regularly meditating.

#### Religious needs

The OR of having religious needs was negative in females (compared to males), and for demographic variables, only the oldest age group was more likely to have religious needs. Crisis of meaning, considering oneself religious or spiritual or both (R + S+), believing in an afterlife, considering God as important (both now and when a child), praying, and meditating were all strongly related to having religious needs, although some associations were reduced in multivariate analyses compared to the univariate ones.

#### Existential needs

Female sex, age 65+, being divorced or unmarried, having greater than one chronic disease, having a crisis of meaning, considering oneself religious, spiritual, or both (R+ S+), believing in an afterlife, finding God important now and during childhood, praying, and meditating, all predicted a greater likelihood of having existential needs. In contrast, a lower likelihood of having existential needs was associated with medium SES, better self-rated physical health, greater well-being, and more satisfaction with life.

#### Generativity needs

After adjusting for covariates, a greater likelihood of having generativity needs was associated with female sex, higher SES, being divorced, having meaning in life and a crisis of meaning, considering oneself religious or spiritual or both (R + S+), finding God important during childhood, praying, and meditating. Better self-rated physical health and higher well-being were associated with fewer generativity needs.

#### Inner peace needs

In the fully adjusted model, female sex, high SES, meaning in life, having a crisis of meaning, considering oneself religious or spiritual or both (R + S+), finding God important during childhood, praying, and meditating were all positively associated with having inner peace needs. In contrast, older age, greater well-being, and higher satisfaction were associated with fewer inner peace needs.

## Discussion

This is the largest study to examine spiritual needs to date, with 26,678 respondents from a random sample of 104,137 adult Danes. The findings shed light on the spiritual needs of this post-secular population while identifying associations between spiritual needs and demographic characteristics and measures of self-rated health, well-being, life satisfaction, meaning in life, as well as spiritual beliefs and practices. Four in five Danes reported some form of spiritual need in the past month (overall SpNQ score was 0.71, with 81.9% reporting at least one strong or very strong spiritual need). Danes scored highest on inner peace needs (68.7% reported at least one need) and generativity needs (63.3%), with existential (41.5%) and religious needs (17.9%) being less prevalent. Spiritual needs were more frequent among those considering themselves spiritual, religious, or both, and for those who pray or meditate regularly. Furthermore, poorer self-rated health, lower life satisfaction and reduced well-being were associated with greater spiritual needs. These findings highlight the salience of spirituality and of spiritual needs in this post-secular population, underlining that the spiritual and religious are by no means irrelevant in Danish society. Furthermore, the findings underscore the importance of addressing spiritual needs in healthcare settings, such as through the taking of a short spiritual history as part of a holistic approach to health care.

### Spiritual needs in a post-secular society

Our data provide evidence that, though predominantly non-religious, most Danes experience spiritual needs. In contrast to spiritual needs in more religious settings, Danes largely experience non-religious spiritual needs, including needs for generatively (i.e., influencing others for good), inner peace, and existential needs (i.e., having meaning in one's life). The prevalence of these needs suggests that they should be assessed as part of a holistic understanding of well-being within healthy and ill persons. Furthermore, they suggest interventions addressing these domains may aid in addressing these needs and influencing well-being; research is needed in this regard. Notably, though Denmark is largely non-religious, almost one in five experience religious needs, suggesting that assessments and interventions in post-secular settings should also include attention to religious needs if present.

Other studies in similar and different samples and settings have used the SpNQ with similar findings, although there are also points of distinction. The following comparisons should be considered in light of the developmental stage in each country and with regard to the importance and influence of religion. As such, the implications for spiritual care that this paper suggest may be different in each of these contexts. Table 4 reports data from samples from different countries which we, for simplicity, have categorised traditionally as ‘secular’ or ‘religious’ (although this distinction has its limitations, as mentioned earlier).^39^, ^40^, ^41^, ^42^, ^43^, ^44^, ^45^ It is noteworthy that the Danish sample scored lowest on religious needs, while the highest level was observed in Poland, comprised largely of a Catholic population. In the Danish sample, existential needs were similar in frequency, except for cancer patients from Lithuania and chronically ill patients from Poland, who scored the highest. In all samples, inner peace needs were highest except in cancer and chronic illness patients from Germany and the elderly from Poland. Also, generativity needs were relatively high in the Danish sample and highest in chronically ill patients from Poland and healthy but stressed mothers of children with Down syndrome from Lithuania. The Danish distribution pattern of spiritual needs, somewhat surprisingly, is similar to Lithuania, although Danes score substantially lower in all dimensions. These patterns of spiritual needs suggest the intensity and type of spiritual needs depend on cultural and religious factors on the one hand, and personal factors (e.g., personal stressors such as illness) on the other.

**Table 4:** Spiritual needs (measured with the spnq with similar factors) reported in external studies ranked by spnq mean score.

Country	Culture	Sample	N	Age^a^	Sex^b^	Spnq mean score	Sample mean score per dimensions of needs			
							Religious	Existential	Generativity	Inner peace
Poland^39^	Religious	Chronic illness patients	275	56	74	1.57	1.59	1.31	1.75	2.03
Portugal^40^^,^^41^	Religious	Dementia	176	77	50	1.25	1.20	0.96	1.39	1.43
Lithuania^42^^,^^c^	Religious	Cancer patients	247	67	58	N/A	1.26	1.33	1.64	1.75
Lithuania^43^	Religious	Healthy mothers of children with Down syndrome	203	44	100	1.14	0.68	0.79	1.52	1.95
Germany^d^	Secular	Cancer patients	381	61	64	1.14	0.93	0.83	1.62	1.47
Germany^44^^,^^45^	Secular	Palliative cancer patients	118	63	58	1.12	0.95	0.78	1.49	1.58
Poland^45^	Religious	Elderly in retirement homes	292	74	63	1.10	1.32	0.62	1.34	1.27
Germany^45^	Secular	Chronic illness patients	855	59	68	1.04	0.72	0.77	1.63	1.35
Germany^40^^,^^45^	Secular	Elderly in retirement homes	345	84	77	0.94	1.08	0.50	1.16	1.21
Italy^45^	Religious	Elderly in retirement homes	164	71	54	0.88	0.90	0.53	1.03	1.21
Germany^41^^,^^44^	Secular	Healthy mothers with sick children	125	31	100	0.78	0.64	0.37	0.92	1.45
*Denmark*^42^	*Secular*	*Random selected Danes*	*23,836*	*55*	*55*	*0.71*	*0.29*	*0.54*	*1.08*	*1.21*

Current study in *italic*.

a Mean age in years.

b Sex as % female.

c Although overall score was N/A it has been ranked with the other Lithuanian study.

d Combined dataset.

### Predictors of spiritual needs

In our multivariate analysis, spiritual beliefs and practices, health, and well-being variables, meaning assessments, and sociodemographic variables influenced the likelihood of reporting spiritual needs. Among religious and spiritual variables, considering oneself religious, spiritual, or both (R+ S+), believing in an afterlife, praying, and meditating, all yielded rather strong positive ORs for having spiritual needs and were generally the most important predictors of spiritual needs across all dimensions. In addition, self-reported poorer health, worse life satisfaction, and less overall well-being were associated with greater spiritual needs. Though the directionality of these associations is unclear, the fact that these self-reported well-being metrics track with spiritual needs suggests that holistic approaches to well-being require attention to spiritual needs. Assessments of meaning also predicted spiritual needs. The association of a crisis of meaning (e.g., feeling that life is empty or without purpose) with greater spiritual needs may reflect how threats to, or deficiencies in, personal meaning result in needs for spiritual and existential resources. Notably, the measure of overall meaning was also associated with greater spiritual needs. One possible hypothesis is that individuals who report greater life meaning are those who more readily acknowledge spiritual needs and seek to address them (resulting in greater self-reported meaning and self-reported needs).^12^ Further research should seek to elucidate these relationships.

Among demographic predictors, complex relationships with spiritual needs and their dimensions were noted. For example, increasing age was positively associated with having more spiritual needs across all dimensions except for inner peace, for which being in the youngest age group exhibited the strongest OR. One possible hypothesis is that with increasing age, thoughts on death and dying and reflecting on meaning in life become increasingly prevalent, resulting in greater existential, generativity, and religious needs. Meanwhile, younger generations may be struggling more with inner peace needs as they face internal identity struggles, as suggested by Erik Erikson's stages of psychosocial development,^46^ potentially together with external social stressors (e.g., Covid-19 pandemic, during which the survey was administered). Overall, sociodemographic predictors of spiritual needs point to the role of personal and social contexts on the types of spiritual needs experienced. Further research is needed to understand the complex interplays of life stage, education, and social contexts on spiritual needs.

### Clinical implications

The frequency of spiritual needs and the aforementioned predictors of spiritual needs point to the need for holistic clinical care contexts to acknowledge and, where required, address the spiritual needs of patients. Attention to spiritual needs can readily be integrated into care by health care professionals through the routine taking of a short spiritual history. Our findings suggest that a few items would likely capture much information regarding whether the patient would potentially experience spiritual needs. Administering 2–4 questions requires only a few minutes in the clinical setting (where lack of time is often a primary barrier to obtaining spiritual histories) and can be readily integrated into patient intake assessments. The positive impact of administering these questions was underscored in our recent report on the development of the EXICODE questionnaire.^32^ Several participants reported that being asked questions regarding their existential and spiritual well-being was a positive and engaging experience.^32^ This finding has been corroborated by the results of a randomised trial of spiritual history-taking in the oncology care setting. The integration of spiritual histories resulted in better patient quality of life and greater sense of interpersonal care from the clinician.^47^ Consequently, taking a spiritual history might be clinically relevant as it may enable the administration of spiritual care and help bring spiritual needs to light, and also, simply taking that spiritual history might be a basic spiritual care intervention that improves the well-being of patients as it signals care and attention to the whole person. A spiritual history can be obtained with single questions or with more advanced tools. “Are religion or spirituality important to you in thinking about health and illness or at other times?” is an example of a singular probe recommended by Balboni et al. (2022).^14^ The FICA spiritual history tool,^48^ and the shortened Spiritual Needs Screener are other examples.^49^ These needs may then be further elaborated by other caregivers or quantified through standardised instruments (such as the more comprehensive SpNQ^30^ or other measures^27^).

Other examples of interventions related to spiritual needs could be courses for clinicians aimed at enhancing their spiritual care competencies,^47^^,^^50^, ^51^, ^52^ or interventions aiming at promoting forgiveness.^53^ Importantly, spiritual care can be provided despite the provider's beliefs—religious, spiritual, atheist, or something else. Nevertheless, the clinical importance of the present study's findings needs further explanation. For example, the nature of the associations of spiritual needs with well-being is not fully understood. Future longitudinal studies are needed to help determine the causal relationships between spiritual needs and physical, mental, social, and spiritual health outcomes. Randomised controlled trials are also required to examine the efficacy of spiritual care interventions on clinical outcomes.

### Limitations

This study has several limitations that affect the generalizability of the findings. First, this was a cross-sectional study, and as such, direction of causation for the associations cannot be determined. Second, the response rate was 25.6%, with non-responders tending to be younger, less educated, have lower income, and be either students or unmarried. This response rate is similar to other population-based studies in Denmark.^54^^,^^55^ Surprisingly, those with chronic diseases and people who were relatively less religious than the general Danish population were more prevalent among respondents. Third, given the taboo surrounding spiritual matters in Denmark, a self-report bias in the survey data is realistic but would most likely underestimate the needs experienced, making our estimates conservative. Fourth, we did not have data on height, weight, or lifestyle factors (e.g., smoking or alcohol). However, we regard this limitation as being of minor importance since we examined SES and chronic diseases (both are associated with lifestyle factors). The potential association of, e.g., smoking or alcohol consumption with spiritual needs could be positive or negative in this Danish sample. Be that as it may, spiritual needs in the present study were associated with religious participation and faith, which have been shown to reduce smoking and alcohol consumption.^56^ Fifth, as always, other statistical approaches and analyses could have been chosen, e.g., multiple imputations for missing values or weighting. Such an analytical approach would be principally different and require other assumptions.

Concerning strengths, first, our sample was large compared to other studies of spiritual needs, enabling unique insights. Second, the use of detailed national Danish registers further strengthens the study, as it provides detailed administrative data collected without regard to the research question. Third, the measures used in this study all had documented acceptable psychometric properties. Fourth, we conducted both linear and logistic regressions to examine the sensitivity of our results.

### Conclusion

This is the largest study of spiritual needs to date. We measured spiritual needs in a sample of randomly selected adult Danes. The results underscore the prevalence of spiritual needs in Danish society, which we term a ‘post-secular society’. In total, 81.9% of Danes reported at least one spiritual need in the past month. Needs related to finding inner peace was the most prevalent, followed by generativity, existential, and religious needs. Taking a spiritual history with a few relevant questions is the beginning of addressing spiritual needs in clinical settings. Our findings point to the complex roles that religious, spiritual, demographic, and health factors play in determining the frequency and types of spiritual needs experienced. The results also point to the need for greater attention to spiritual needs within healthcare settings and the need for future research to guide spiritual care interventions as part of a holistic approach to health care in post-secular cultures.

## Contributors

Conceptualisation: TAS, SW, AB, JS, NCH; Data curation: TAS, SW; Formal analysis: TAS, SW; Funding acquisition: TAS, SW, JS, NCH; Project administration: TAS, NCH; Supervision: SW, JS, NCH; Validation: TAS, SW; Visualisation: TAS, SW; Writing—original draft: TAS; Writing—review & editing: TAS, SW, AB, HGK, TAB, TJV, JS, NCH. All authors approved the final manuscript.

## Data sharing statement

Due to data sensitivity and legislation, data are not openly available. The data comes from the EXICODE study which comprises a vast dataset, and while not publicly available, the authors invite all interested parties to reach out for collaborations.

## Declaration of interests

TAS: none.

SW: none.

AB: received support for attending meetings and/or travel; International Society of Spirituality and Health (IGGS) - Lodging and conferences fees for the 2022 annual meeting were paid by the IGGS and European Conference for Religion, Spirituality and Heath (EC RSH) - As member of the scientific community my conference fees for the 2022 conference were not charged by the EC RSH.

HGK: none.

TAB: none.

TV: TV receives licensing fees from Flerish Inc. and Flourishing Metrics.

JS: JS reports grants from EU, grants from Danish Research Council and from other funds outside the submitted work. JS has participated in scientific advisory boards for Novo Nordic, Roche, Astra-Zeneca, GlaxoSmith Kline Pharma. JS is editor for Promedicin. dk.

NCH: none.

## Appendix A Supplementary data

Supplementary appendices
