# Supplementary material for: Spiritual needs in Denmark: a population-based cross-sectional survey linked to Danish national registers
Source: Lancet Reg Health Eur. 2023 Mar 12;28:100602. doi: 10.1016/j.lanepe.2023.100602 (PMC10173272; doi:10.1016/j.lanepe.2023.100602)

APPENDICES FOR:

Spiritual needs in Denmark: a population-based cross-sectional survey linked to Danish national registers

By T. A. Stripp et al.

Contents

APPENDIX 1 3
APPENDIX 2 4
APPENDIX 2.1 4
APPENDIX 2.2 5
APPENDIX 2.3 6
APPENDIX 2.4 7
APPENDIX 2.5 8
APPENDIX 3 9
APPENDIX 3.1 9
APPENDIX 3.2 11
APPENDIX 3.3 13
APPENDIX 3.4 15
APPENDIX 4 17
APPENDIX 4.1 17
APPENDIX 4.2 18
APPENDIX 4.3 19
APPENDIX 4.4 20
APPENDIX 5 21
APPENDIX 5.1 21
APPENDIX 5.2 23
APPENDIX 5.3 25
APPENDIX 5.4 27
APPENDIX 5.5 29
APPENDIX 6 31
APPENDIX 6.1 31
APPENDIX 6.2 32
APPENDIX 6.3 33
APPENDIX 6.4 34
APPENDIX 6.5 35

APPENDIX 1

DEMOGRAPHIC VARIABLES OF PARTICIPANTS INVITED TO THE SURVEY BY RESPONDENTS AND NON-RESPONDENTS (N = 104,137).

		ALL	RESPONDENTS	NON-RESPONDENTS
**TOTAL**		104137 (100.0)	26678 (25.6)	77459 (74.4)
**SEX**	Male	51980 (100.0)	11827 (22.8)	40153 (77.2)
	Female	52157 (100.0)	14851 (28.5)	37306 (71.5)
**AGE, YEARS**	18-25	14703 (100.0)	1710 (11.6)	12993 (88.4)
	26-35	18007 (100.0)	2446 (13.6)	15561 (86.4)
	36-45	16450 (100.0)	3042 (18.5)	13408 (81.5)
	46-55	18708 (100.0)	5072 (27.1)	13636 (72.9)
	56-65	16484 (100.0)	6088 (36.9)	10396 (63.1)
	66-75	13009 (100.0)	5826 (44.8)	7183 (55.2)
	75+	6776 (100.0)	2494 (36.8)	4282 (63.2)
**EDUCATION, YEARS**	7-	24433 (100.0)	4698 (19.2)	19735 (80.8)
	12-	42472 (100.0)	10998 (25.9)	31474 (74.1)
	15-	33096 (100.0)	10473 (31.6)	22623 (68.4)
	Missing/Unknown	4136 (100.0)	509 (12.3)	3627 (87.7)
**INCOME, RELATIVE TO AGE**	Lower	31201 (100.0)	6336 (20.3)	24865 (79.7)
	Middle	34871 (100.0)	9277 (26.6)	25594 (73.4)
	Upper	36128 (100.0)	10866 (30.1)	25262 (69.9)
	Unknown/missing	1937 (100.0)	199 (10.3)	1738 (89.7)
**LIVING STATUS**	Living alone	34349 (100.0)	7725 (22.5)	26624 (77.5)
	Living with someone	67851 (100.0)	18754 (27.6)	49097 (72.4)
	Missing/unknown	1937 (100.0)	199 (10.3)	1738 (89.7)
**REGION OF RESIDENCE**	North	10436 (100.0)	2606 (25.0)	7830 (75.0)
	Mid	23501 (100.0)	6068 (25.8)	17433 (74.2)
	South	21310 (100.0)	6065 (28.5)	15245 (71.5)
	Capitol	32519 (100.0)	7762 (23.9)	24757 (76.1)
	Zealand	14434 (100.0)	3978 (27.6)	10456 (72.4)
	Unknown/missing	1937 (100.0)	199 (10.3)	1738 (89.7)
**WORKING STATUS**	Working	50927 (100.0)	12179 (23.9)	38748 (76.1)
	Student benefit	6998 (100.0)	1000 (14.3)	5998 (85.7)
	Other public benefit	25700 (100.0)	5644 (22.0)	20056 (78.0)
	Public and private pension(s)	19549 (100.0)	7773 (39.8)	11776 (60.2)
	Unknown/missing	963 (100.0)	82 (8.5)	881 (91.5)
**CIVIL STATUS**	Married	48085 (100.0)	15256 (31.7)	32829 (68.3)
	Widow/er	3975 (100.0)	1437 (36.2)	2538 (63.8)
	Divorced	10979 (100.0)	3297 (30.0)	7682 (70.0)
	Unmarried	39161 (100.0)	6489 (16.6)	32672 (83.4)
	Missing/Unknown	1937 (100.0)	199 (10.3)	1738 (89.7)
**CHRONIC DISEASE**	No	84242 (100.0)	20801 (24.7)	63441 (75.3)
	Yes, one	17819 (100.0)	5210 (29.2)	12609 (70.8)
	More than one	2076 (100.0)	667 (32.1)	1409 (67.9)

All groups were statistically significantly different at p<0.001, based on Chi-squared tests excluding missing values.

APPENDIX 2

DISTRIBUTION OF SPIRITUAL NEEDS SCORES.

APPENDIX 2.1

DISTRIBUTION (INTERQUARTILE RANGE (IQR)) OF OVERALL SPIRITUAL NEEDS SCORES OF ADULT DANES BY DEMOGRAPHIC VARIABLES (N = 23,826).

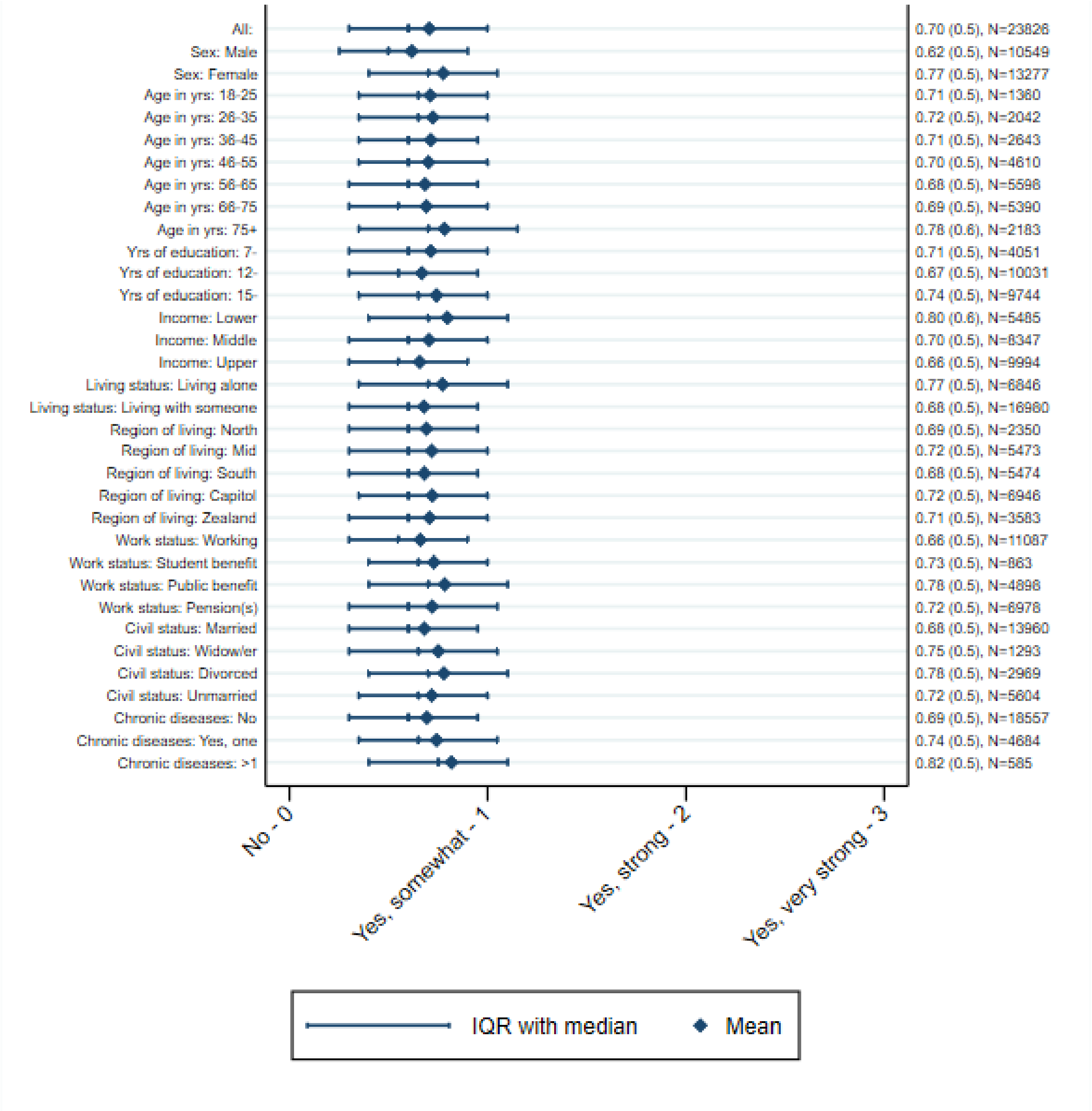

APPENDIX 2.2

DISTRIBUTION (INTERQUARTILE RANGE (IQR)) OF RELIGIOUS NEEDS SCORES OF ADULT DANES BY DEMOGRAPHIC VARIABLES (N = 23,826).

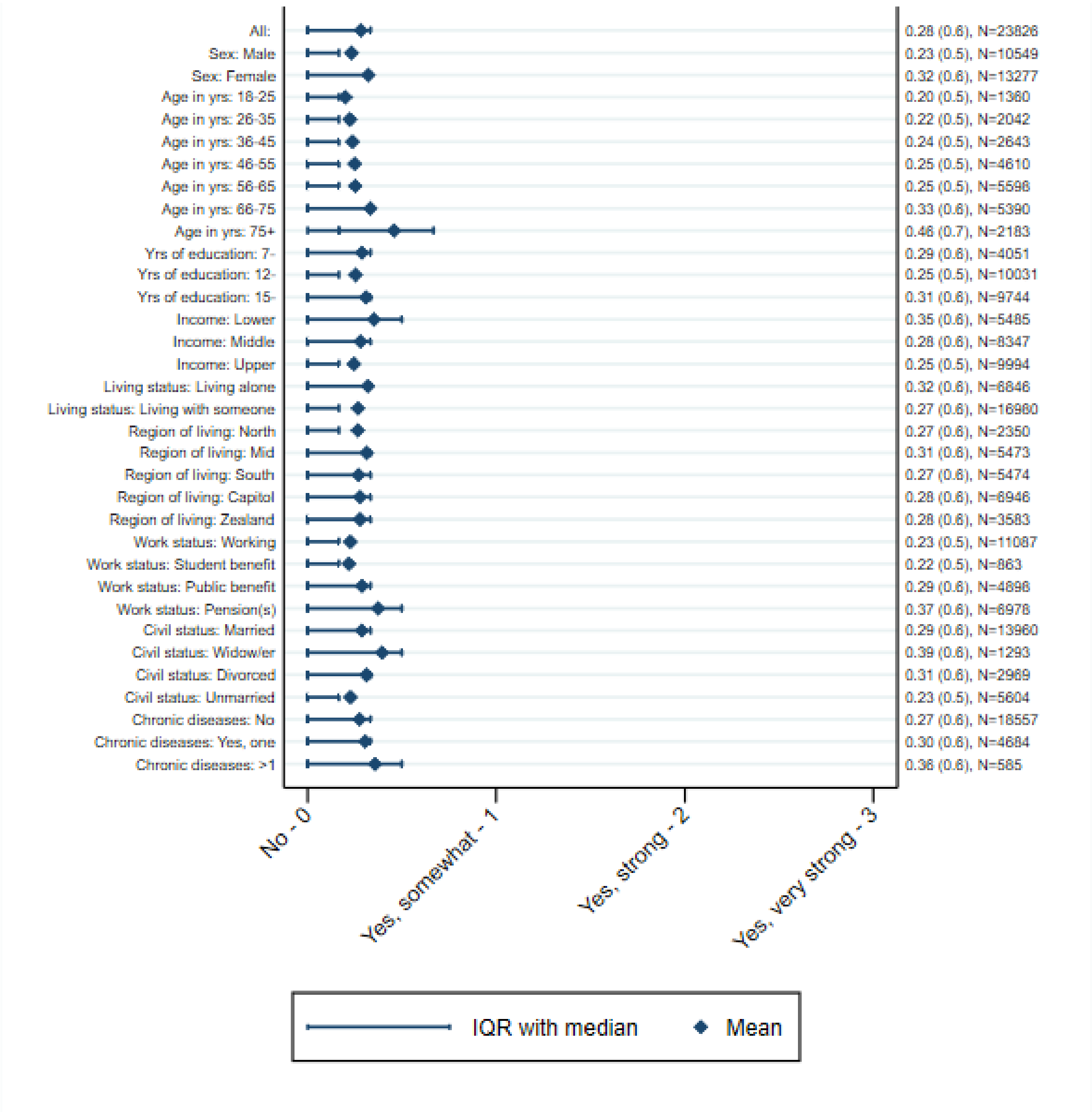

APPENDIX 2.3

DISTRIBUTION (INTERQUARTILE RANGE (IQR)) OF EXISTENTIAL NEEDS SCORES OF ADULT DANES BY DEMOGRAPHIC VARIABLES (N = 23,826).

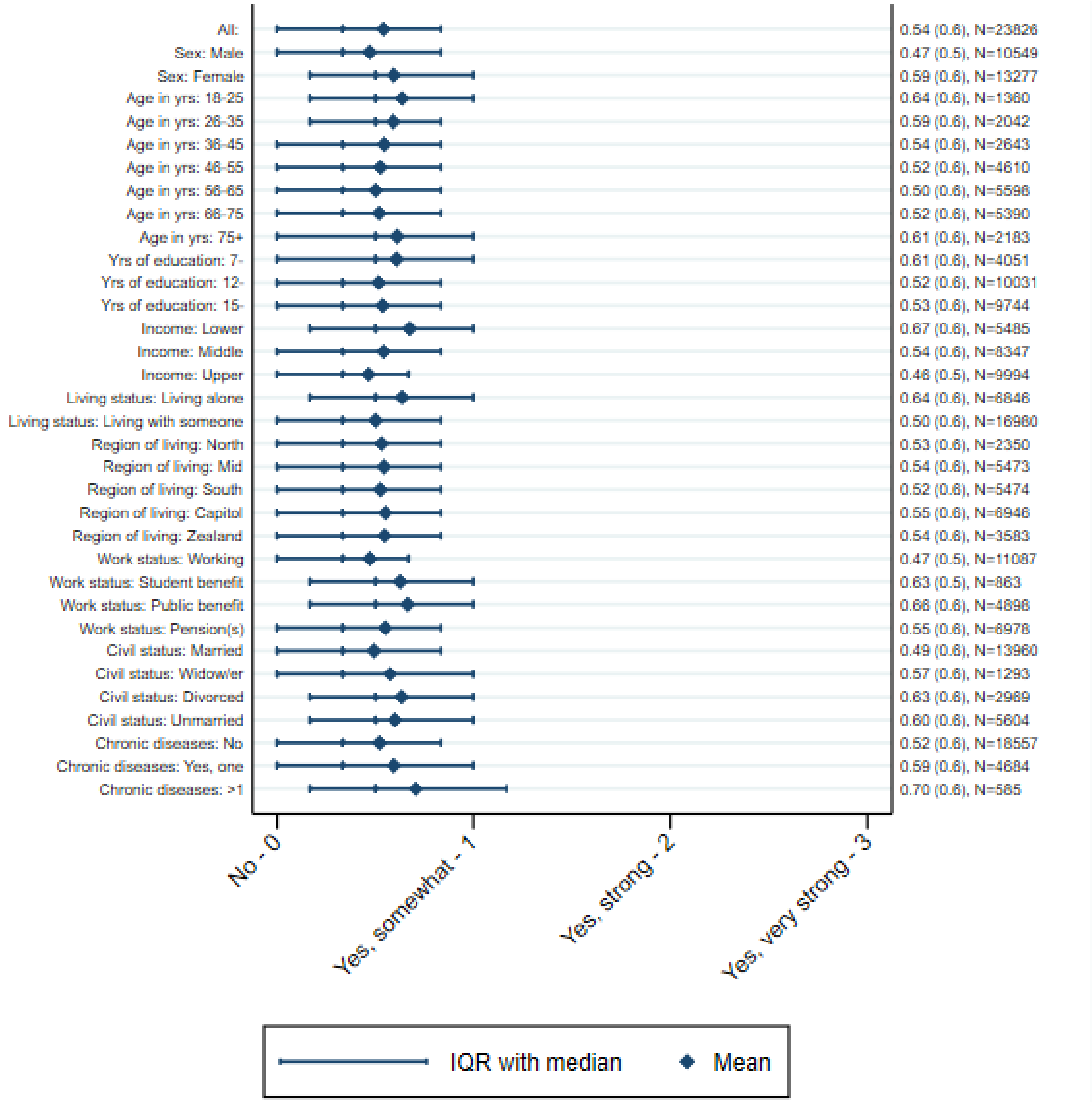

APPENDIX 2.4

DISTRIBUTION (INTERQUARTILE RANGE (IQR)) OF GENERATIVITY NEEDS SCORES OF ADULT DANES BY DEMOGRAPHIC VARIABLES (N = 23,826).

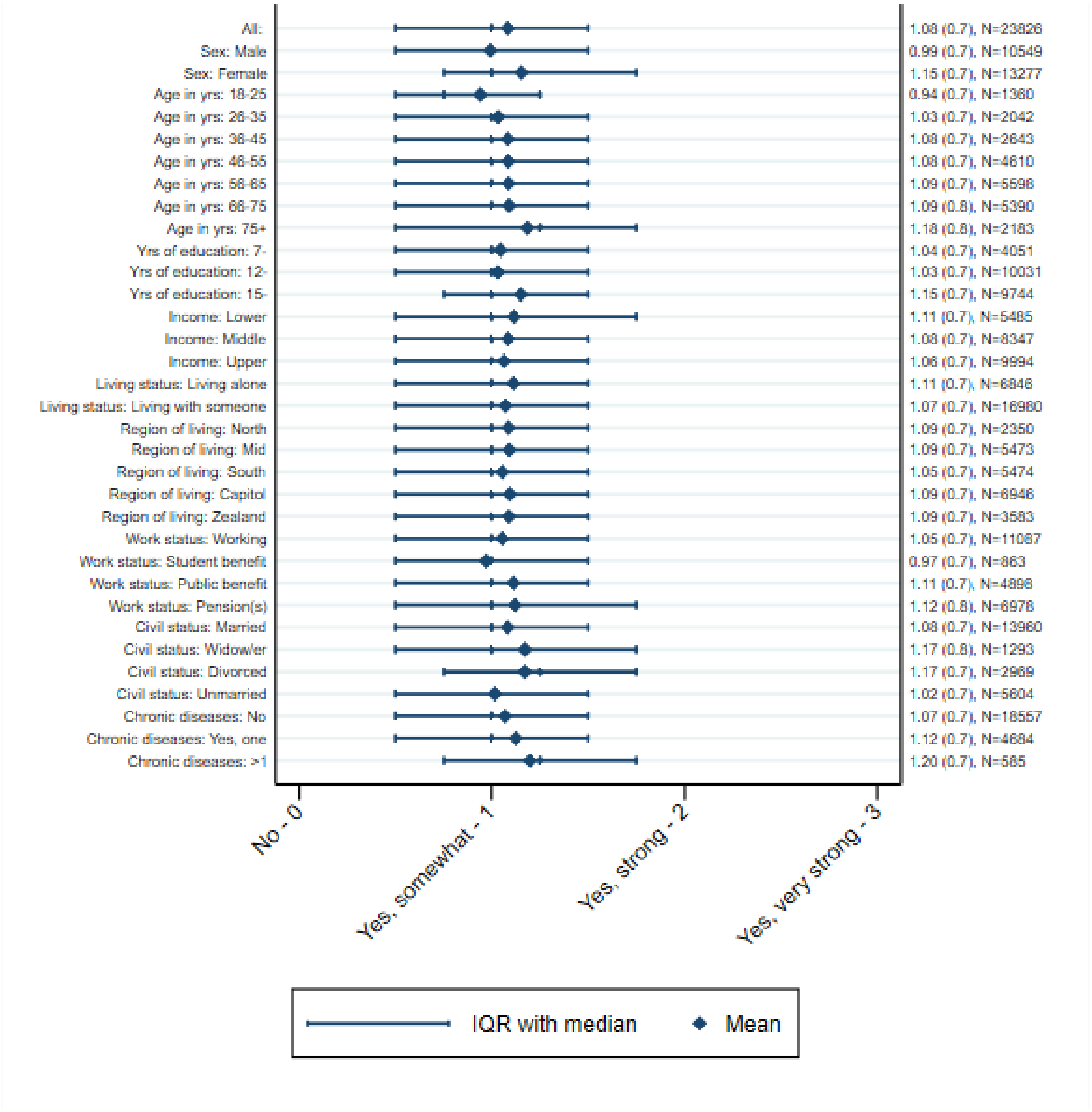

APPENDIX 2.5

DISTRIBUTION (INTERQUARTILE RANGE (IQR)) OF INNER PEACE NEEDS SCORES OF ADULT DANES BY DEMOGRAPHIC VARIABLES (N = 23,826).

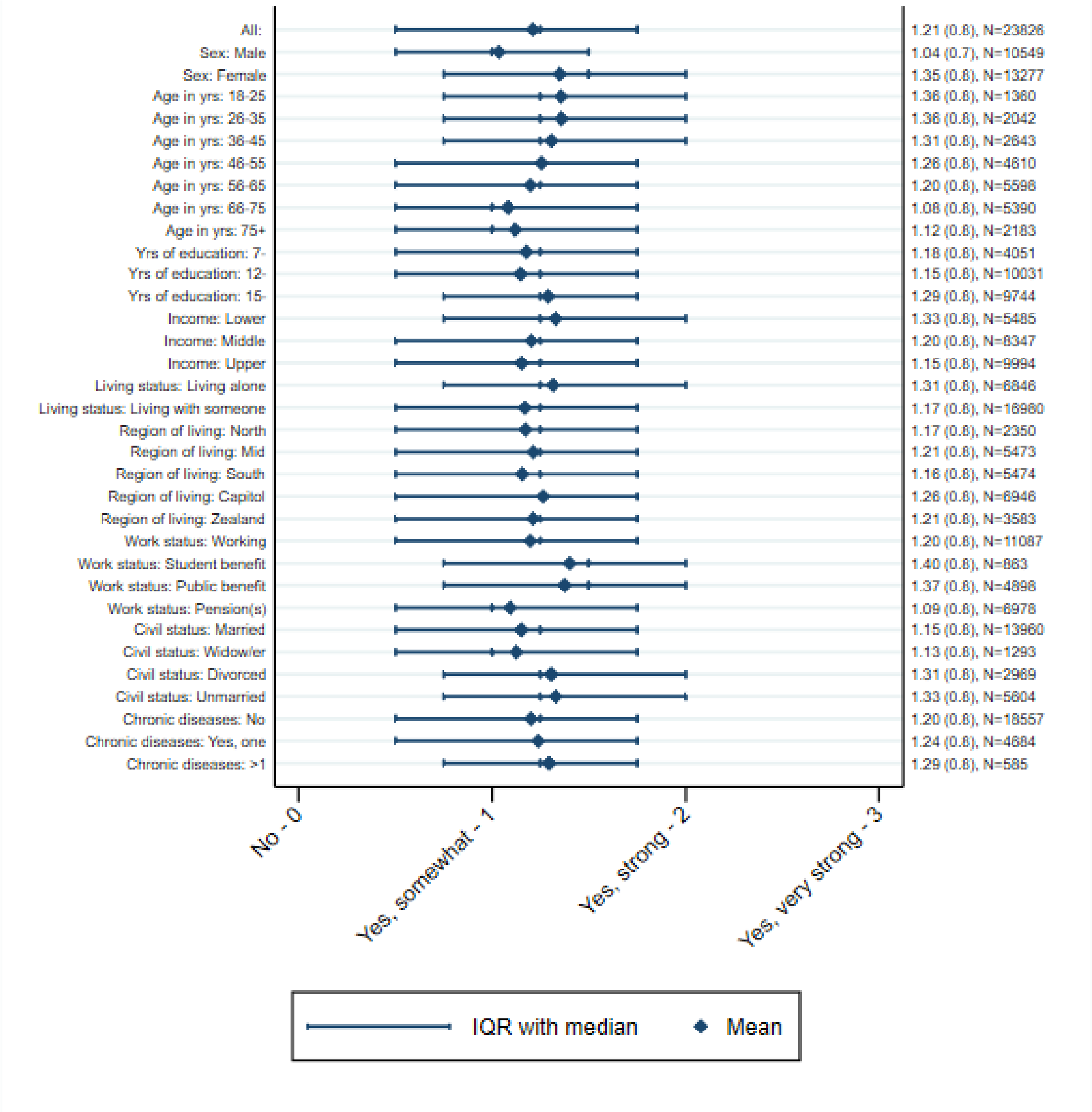

APPENDIX 3

UNI- AND MULTIVARIABLE LOGISTIC REGRESSION MODELS FOR EACH SPNQ DIMENSION.

APPENDIX 3.1

ODDS RATIOS OF HAVING RELIGIOUS NEEDS BY DEMOGRAPHIC VARIABLES AND SPIRITUAL BELIEFS AND PRACTICES (N=23,826). MODEL 1 = RELIGIOUS NEEDS AS DEPENDENT VARIABLE; DEMOGRAPHIC VARIABLES AS INDEPENDENT VARIABLES. MODEL 2 = RELIGIOUS NEEDS AS DEPENDENT VARIABLE; DEMOGRAPHIC VARIABLES AND SURVEYED OUTCOMES AS INDEPENDENT VARIABLES.

	**UNIVARIATE REGRESSIONS**		**MODEL 1 (R^2=0.023)**			**MODEL 2 (R^2=0.421)**		
VARIABLES	**ODDS RATIO**	P-VAL	**ODDS RATIO**	P-VAL	P-VAL	**ODDS RATIO**	P-VAL	P-VAL
**CONSTANT**			0.224 (0.191;0.262)	<0.001		0.058 (0.040;0.084)	<0.001	
**MALE**	Ref	Ref	Ref			Ref	.	
**FEMALE**	1.473 (1.381;1.572)	<0.001	1.527 (1.429;1.632)	<0.001		0.840 (0.765;0.923)	<0.001	
**AGE 18-45**	Ref	Ref	Ref		<0.001 †	Ref	.	<0.001 †
**AGE 46-65**	1.206 (1.108;1.314)	<0.001	1.153 (1.050;1.268)	0.003		1.008 (0.889;1.143)	0.901	
**AGE 65+**	1.953 (1.793;2.128)	<0.001	1.862 (1.681;2.062)	<0.001		1.620 (1.407;1.865)	<0.001	
**LOW SES**	Ref	Ref	Ref		0.030†	Ref	.	0.208†
**MEDIUM SES**	0.858 (0.786;0.937)	<0.001	0.919 (0.840;1.005)	0.065		0.899 (0.796;1.016)	0.089	
**HIGH SES**	0.833 (0.771;0.900)	<0.001	0.896 (0.826;0.972)	0.009		0.965 (0.862;1.081)	0.539	
**LIVING ALONE**	Ref	Ref	Ref			Ref	.	
**LIVING WITH SOMEONE**	0.774 (0.724;0.828)	<0.001	0.710 (0.639;0.790)	<0.001		0.926 (0.803;1.068)	0.292	
**MARRIED**	Ref	Ref	Ref		<0.001 †	Ref	.	0.243†
**WIDOW(ER)**	1.636 (1.442;1.856)	<0.001	0.865 (0.737;1.016)	0.077		1.080 (0.866;1.346)	0.496	
**DIVORCED**	1.204 (1.096;1.322)	<0.001	0.905 (0.799;1.026)	0.118		0.948 (0.801;1.123)	0.538	
**UNMARRIED**	0.763 (0.703;0.828)	<0.001	0.760 (0.678;0.852)	<0.001		0.889 (0.764;1.034)	0.126	
**NO CHRONIC DISEASE**	Ref	Ref	Ref		0.382†	Ref	.	0.780†
**1 CHRONIC DISEASE**	1.170 (1.083;1.264)	<0.001	1.034 (0.955;1.120)	0.406		0.985 (0.883;1.099)	0.786	
**>1 CHRONIC DISEASE**	1.515 (1.258;1.824)	<0.001	1.123 (0.929;1.358)	0.231		1.085 (0.836;1.408)	0.540	
**SELF-RATED PHYSICAL HEALTH**	0.975 (0.961;0.989)	<0.001				0.999 (0.974;1.024)	0.932	
**WELL-BEING (MENTAL)**	0.996 (0.995;0.998)	<0.001				0.998 (0.995;1.001)	0.168	
**SATISFACTION WITH LIFE**	0.995 (0.993;0.996)	<0.001				0.994 (0.990;0.999)	0.009	
**MEANING IN LIFE**	0.985 (0.958;1.012)	0.273				0.975 (0.926;1.026)	0.326	
**CRISIS OF MEANING**	1.162 (1.132;1.193)	<0.001				1.291 (1.229;1.356)	<0.001	
**R/S* : NONE/DON'T KNOW**	Ref	Ref				Ref	.	<0.001 †
**RELIGIOUS**	14.019 (12.860;15.282)	<0.001				3.643 (3.250;4.083)	<0.001	
**SPIRITUAL**	8.280 (7.420;9.240)	<0.001				4.183 (3.641;4.806)	<0.001	
**RELIGIOUS AND SPIRITUAL**	22.602 (20.028;25.507)	<0.001				5.216 (4.465;6.094)	<0.001	
**BELIEF IN AFTERLIFE**	1.584 (1.527;1.643)	<0.001				1.132 (1.072;1.196)	<0.001	

**GOD IMPORTANT TODAY**	17.547 (16.246;18.951)	<0.001				3.394 (3.030;3.802)	<0.001	
**GOD IMPORTANT AS CHILD**	6.193 (5.783;6.631)	<0.001				1.435 (1.295;1.591)	<0.001	
**PRAYING OFTEN OR REGULARLY**	22.618 (20.900;24.477)	<0.001				6.173 (5.588;6.819)	<0.001	
**MEDITATING OFTEN OR REGULARLY**	4.423 (4.113;4.757)	<0.001				2.030 (1.823;2.260)	<0.001	

† = Wald test estimate for categorical variables.

APPENDIX 3.2

ODDS RATIOS OF HAVING EXISTENTIAL NEEDS BY DEMOGRAPHIC VARIABLES AND SPIRITUAL BELIEFS AND PRACTICES (N=23,826). MODEL 1 = EXISTENTIAL NEEDS AS DEPENDENT VARIABLE; DEMOGRAPHIC VARIABLES AS INDEPENDENT VARIABLES. MODEL 2 = EXISTENTIAL NEEDS AS DEPENDENT VARIABLE; DEMOGRAPHIC VARIABLES AND SURVEYED OUTCOMES AS INDEPENDENT VARIABLES.

	**UNIVARIATE REGRESSIONS**		**MODEL 1 (R^2=0.019)**			**MODEL 2 (R^2=0.158)**		
**VARIABLES**	**ODDS RATIO**	**P-VAL**	**ODDS RATIO**	**P-VAL**	**P-VAL**	**ODDS RATIO**	**P-VAL**	**P-VAL**
**CONSTANT**			0.923 (0.818;1.042)	0.195		2.880 (2.245;3.695)	<0.001	
**MALE**	Ref	Ref	Ref			Ref	.	
**FEMALE**	1.481 (1.406;1.559)	<0.001	1.434 (1.360;1.512)	<0.001		1.241 (1.169;1.319)	<0.001	
**AGE 18-45**	Ref	Ref	Ref		<0.001 †	Ref	.	<0.001 †
**AGE 46-65**	0.763 (0.716;0.813)	<0.001	0.833 (0.776;0.895)	<0.001		0.961 (0.886;1.042)	0.337	
**AGE 65+**	0.831 (0.777;0.889)	<0.001	0.931 (0.859;1.010)	0.087		1.223 (1.114;1.341)	<0.001	
**LOW SES**	Ref	Ref	Ref		<0.001 †	Ref	.	0.127†
**MEDIUM SES**	0.762 (0.709;0.819)	<0.001	0.828 (0.769;0.891)	<0.001		0.919 (0.846;0.997)	0.043	
**HIGH SES**	0.661 (0.620;0.704)	<0.001	0.756 (0.707;0.809)	<0.001		0.958 (0.888;1.034)	0.273	
**LIVING ALONE**	Ref	Ref	Ref			Ref	.	
**LIVING WITH SOMEONE**	0.674 (0.637;0.713)	<0.001	0.840 (0.774;0.912)	<0.001		1.069 (0.975;1.173)	0.153	
**MARRIED**	Ref	Ref	Ref		<0.001 †	Ref	.	0.002†
**WIDOW(ER)**	1.298 (1.158;1.455)	<0.001	0.940 (0.819;1.079)	0.379		1.055 (0.905;1.229)	0.495	
**DIVORCED**	1.535 (1.417;1.662)	<0.001	1.264 (1.144;1.397)	<0.001		1.222 (1.094;1.366)	<0.001	
**UNMARRIED**	1.472 (1.384;1.567)	<0.001	1.214 (1.115;1.321)	<0.001		1.145 (1.042;1.259)	0.005	
**NO CHRONIC DISEASE**	Ref	Ref	Ref		<0.001 †	Ref	.	0.067†
**1 CHRONIC DISEASE**	1.240 (1.163;1.322)	<0.001	1.199 (1.123;1.280)	<0.001		1.050 (0.976;1.130)	0.187	
**>1 CHRONIC DISEASE**	1.818 (1.538;2.149)	<0.001	1.658 (1.398;1.967)	<0.001		1.218 (1.008;1.472)	0.041	
**SELF-RATED PHYSICAL HEALTH**	0.804 (0.794;0.815)	<0.001				0.926 (0.911;0.942)	<0.001	
**WELL-BEING (MENTAL)**	0.969 (0.968;0.971)	<0.001				0.986 (0.984;0.988)	<0.001	
**SATISFACTION WITH LIFE**	0.960 (0.959;0.962)	<0.001				0.988 (0.985;0.991)	<0.001	
**MEANING IN LIFE**	0.692 (0.675;0.709)	<0.001				1.025 (0.990;1.061)	0.165	
**CRISIS OF MEANING**	1.776 (1.729;1.824)	<0.001				1.470 (1.418;1.524)	<0.001	
**R/S* : NONE/DON'T KNOW**	Ref	Ref				Ref	.	<0.001 †
**RELIGIOUS**	1.849 (1.726;1.981)	<0.001				1.398 (1.276;1.531)	<0.001	
**SPIRITUAL**	3.227 (2.921;3.565)	<0.001				2.105 (1.878;2.360)	<0.001	
**RELIGIOUS AND SPIRITUAL**	3.471 (3.098;3.890)	<0.001				1.910 (1.669;2.187)	<0.001	
**BELIEF IN AFTERLIFE**	1.253 (1.216;1.290)	<0.001				1.062 (1.026;1.099)	<0.001	
**GOD IMPORTANT TODAY**	2.424 (2.269;2.590)	<0.001				1.352 (1.222;1.497)	<0.001	

**GOD IMPORTANT AS CHILD**	2.104 (1.980;2.236)	<0.001				1.420 (1.311;1.537)	<0.001	
**PRAYING OFTEN OR REGULARLY**	2.565 (2.402;2.738)	<0.001				1.371 (1.250;1.503)	<0.001	
**MEDITATING OFTEN OR REGULARLY**	2.863 (2.667;3.074)	<0.001				1.972 (1.815;2.144)	<0.001	

† = Wald test estimate for categorical variables.

APPENDIX 3.3

ODDS RATIOS OF HAVING GENERATIVITY NEEDS BY DEMOGRAPHIC VARIABLES AND SPIRITUAL BELIEFS AND PRACTICES (N=23,826). MODEL 1 = GENERATIVITY NEEDS AS DEPENDENT VARIABLE; DEMOGRAPHIC VARIABLES AS INDEPENDENT VARIABLES. MODEL 2 = GENERATIVITY NEEDS AS DEPENDENT VARIABLE; DEMOGRAPHIC VARIABLES AND SURVEYED OUTCOMES AS INDEPENDENT VARIABLES.

	**UNIVARIATE REGRESSIONS**		**MODEL 1 (R^2=0.016)**			**MODEL 2 (R^2=0.061)**		
**VARIABLES**	**ODDS RATIO**	**P-VAL**	**ODDS RATIO**	**P-VAL**	**P-VAL**	**ODDS RATIO**	**P-VAL**	**P-VAL**
**CONSTANT**			3.116 (2.670;3.636)	<0.001		1.828 (1.362;2.452)	<0.001	
**MALE**	Ref	Ref	Ref			Ref	.	
**FEMALE**	1.624 (1.520;1.736)	<0.001	1.597 (1.493;1.709)	<0.001		1.369 (1.275;1.470)	<0.001	
**AGE 18-45**	Ref	Ref	Ref		0.003†	Ref	.	0.018†
**AGE 46-65**	1.099 (1.011;1.194)	0.027	1.036 (0.943;1.137)	0.465		1.077 (0.977;1.187)	0.138	
**AGE 65+**	0.940 (0.862;1.025)	0.161	0.900 (0.810;0.999)	0.049		0.958 (0.858;1.071)	0.453	
**LOW SES**	Ref	Ref	Ref		<0.001 †	Ref	.	<0.001 †
**MEDIUM SES**	1.037 (0.948;1.133)	0.426	1.086 (0.992;1.189)	0.076		1.096 (0.998;1.204)	0.054	
**HIGH SES**	1.413 (1.301;1.533)	<0.001	1.496 (1.373;1.630)	<0.001		1.551 (1.418;1.697)	<0.001	
**LIVING ALONE**	Ref	Ref	Ref			Ref	.	
**LIVING WITH SOMEONE**	0.926 (0.860;0.997)	0.042	0.905 (0.814;1.006)	0.064		1.013 (0.909;1.130)	0.810	
**MARRIED**	Ref	Ref	Ref		<0.001 †	Ref	.	<0.001 †
**WIDOW(ER)**	1.144 (0.981;1.334)	0.087	1.070 (0.891;1.285)	0.469		1.175 (0.974;1.417)	0.092	
**DIVORCED**	1.295 (1.160;1.447)	<0.001	1.229 (1.075;1.407)	0.003		1.230 (1.072;1.411)	0.003	
**UNMARRIED**	0.910 (0.841;0.985)	0.019	0.894 (0.802;0.997)	0.044		0.915 (0.818;1.024)	0.123	
**NO CHRONIC DISEASE**	Ref	Ref	Ref		0.021†	Ref	.	0.204†
**1 CHRONIC DISEASE**	1.056 (0.971;1.149)	0.201	1.070 (0.982;1.165)	0.125		1.019 (0.933;1.114)	0.669	
**>1 CHRONIC DISEASE**	1.346 (1.064;1.703)	0.013	1.348 (1.062;1.711)	0.014		1.247 (0.976;1.592)	0.078	
**SELF-RATED PHYSICAL HEALTH**	0.946 (0.931;0.961)	<0.001				0.962 (0.942;0.982)	<0.001	
**WELL-BEING (MENTAL)**	0.991 (0.990;0.993)	<0.001				0.994 (0.991;0.996)	<0.001	
**SATISFACTION WITH LIFE**	0.994 (0.992;0.996)	<0.001				1.002 (0.998;1.005)	0.330	
**MEANING IN LIFE**	1.025 (0.996;1.056)	0.090				1.144 (1.101;1.189)	<0.001	
**CRISIS OF MEANING**	1.151 (1.115;1.189)	<0.001				1.216 (1.164;1.271)	<0.001	
**R/S* : NONE/DON'T KNOW**	Ref	Ref				Ref	.	<0.001 †
**RELIGIOUS**	2.007 (1.814;2.220)	<0.001				1.482 (1.314;1.671)	<0.001	
**SPIRITUAL**	3.196 (2.702;3.780)	<0.001				2.008 (1.684;2.395)	<0.001	
**RELIGIOUS AND SPIRITUAL**	3.913 (3.181;4.814)	<0.001				2.066 (1.655;2.579)	<0.001	
**BELIEF IN AFTERLIFE**	1.145 (1.101;1.190)	<0.001				0.982 (0.943;1.022)	0.374	
**GOD IMPORTANT TODAY**	2.191 (1.979;2.424)	<0.001				1.080 (0.941;1.240)	0.272	
**GOD IMPORTANT AS CHILD**	1.951 (1.784;2.135)	<0.001				1.327 (1.194;1.475)	<0.001	

**PRAYING OFTEN OR REGULARLY**	2.534 (2.283;2.813)	<0.001				1.344 (1.179;1.531)	<0.001		
**MEDITATING OFTEN OR REGULARLY**	3.538 (3.115;4.018)	<0.001				2.321 (2.028;2.656)	<0.001		

† = Wald test estimate for categorical variables.

APPENDIX 3.4

ODDS RATIOS OF HAVING INNER PEACE NEEDS BY DEMOGRAPHIC VARIABLES AND SPIRITUAL BELIEFS AND PRACTICES (N=23,826). MODEL 1 = INNER PEACE NEEDS AS DEPENDENT VARIABLE; DEMOGRAPHIC VARIABLES AS INDEPENDENT VARIABLES. MODEL 2 = INNER PEACE NEEDS AS DEPENDENT VARIABLE; DEMOGRAPHIC VARIABLES AND SURVEYED OUTCOMES AS INDEPENDENT VARIABLES.

	**UNIVARIATE REGRESSIONS**		**MODEL 1 (R^2=0.026)**			**MODEL 2 (R^2=0.111)**		
**VARIABLES**	**ODDS RATIO**	**P-VAL**	**ODDS RATIO**	**P-VAL**	**P-VAL**	**ODDS RATIO**	**P-VAL**	**P-VAL**
**CONSTANT**			4.738 (4.018;5.587)	<0.001		11.540 (8.298;16.049)	<0.001	
**MALE**	Ref	Ref	Ref			Ref	.	
**FEMALE**	1.901 (1.778;2.033)	<0.001	1.819 (1.700;1.948)	<0.001		1.549 (1.440;1.667)	<0.001	
**AGE 18-45**	Ref	Ref	Ref		<0.001 †	Ref	.	<0.001 †
**AGE 46-65**	0.685 (0.626;0.750)	<0.001	0.731 (0.661;0.808)	<0.001		0.859 (0.773;0.956)	0.005	
**AGE 65+**	0.540 (0.492;0.592)	<0.001	0.615 (0.551;0.686)	<0.001		0.785 (0.698;0.882)	<0.001	
**LOW SES**	Ref	Ref	Ref		<0.001 †	Ref	.	<0.001 †
**MEDIUM SES**	0.899 (0.820;0.985)	0.022	0.971 (0.884;1.067)	0.545		1.030 (0.933;1.137)	0.557	
**HIGH SES**	1.080 (0.993;1.174)	0.073	1.225 (1.122;1.337)	<0.001		1.407 (1.281;1.544)	<0.001	
**LIVING ALONE**	Ref	Ref	Ref			Ref	.	
**LIVING WITH SOMEONE**	0.743 (0.688;0.802)	<0.001	0.786 (0.703;0.880)	<0.001		0.940 (0.835;1.058)	0.303	
**MARRIED**	Ref	Ref	Ref		<0.001 †	Ref	.	0.163†
**WIDOW(ER)**	0.987 (0.856;1.137)	0.853	0.817 (0.685;0.974)	0.024		0.915 (0.762;1.100)	0.346	
**DIVORCED**	1.408 (1.263;1.571)	<0.001	1.169 (1.021;1.338)	0.024		1.117 (0.970;1.287)	0.125	
**UNMARRIED**	1.480 (1.359;1.612)	<0.001	1.097 (0.977;1.231)	0.116		1.032 (0.914;1.165)	0.613	
**NO CHRONIC DISEASE**	Ref	Ref	Ref		0.030†	Ref	.	0.579†
**1 CHRONIC DISEASE**	1.037 (0.954;1.128)	0.394	1.072 (0.984;1.168)	0.111		0.957 (0.875;1.048)	0.344	
**>1 CHRONIC DISEASE**	1.238 (0.985;1.556)	0.068	1.306 (1.035;1.648)	0.025		1.044 (0.818;1.333)	0.730	
**SELF-RATED PHYSICAL HEALTH**	0.863 (0.849;0.878)	<0.001				0.977 (0.956;1.000)	0.049	
**WELL-BEING (MENTAL)**	0.971 (0.969;0.973)	<0.001				0.981 (0.978;0.984)	<0.001	
**SATISFACTION WITH LIFE**	0.969 (0.967;0.972)	<0.001				0.991 (0.987;0.995)	<0.001	
**MEANING IN LIFE**	0.834 (0.808;0.862)	<0.001				1.124 (1.079;1.171)	<0.001	
**CRISIS OF MEANING**	1.539 (1.478;1.601)	<0.001				1.325 (1.260;1.393)	<0.001	
**R/S* : NONE/DON'T KNOW**	Ref	Ref				Ref	.	<0.001 †
**RELIGIOUS**	1.599 (1.455;1.758)	<0.001				1.336 (1.189;1.501)	<0.001	
**SPIRITUAL**	5.377 (4.361;6.630)	<0.001				2.861 (2.300;3.559)	<0.001	
**RELIGIOUS AND SPIRITUAL**	4.146 (3.350;5.131)	<0.001				2.055 (1.633;2.587)	<0.001	
**BELIEF IN AFTERLIFE**	1.187 (1.141;1.234)	<0.001				0.981 (0.941;1.022)	0.359	
**GOD IMPORTANT TODAY**	1.943 (1.762;2.142)	<0.001				1.061 (0.924;1.219)	0.400	
**GOD IMPORTANT AS CHILD**	1.879 (1.719;2.054)	<0.001				1.359 (1.220;1.514)	<0.001	

**PRAYING OFTEN OR REGULARLY**	2.348 (2.121;2.599)	<0.001				1.312 (1.151;1.496)	<0.001	
**MEDITATING OFTEN OR REGULARLY**	5.073 (4.383;5.872)	<0.001				3.138 (2.691;3.660)	<0.001	

† = Wald test estimate for categorical variables.

APPENDIX 4

FOREST PLOTS OF MULTIVARIABLE LOGISTIC REGRESSION MODELS.

APPENDIX 4.1

ODDS RATIO FOR HAVING RELIGIOUS NEEDS BY DEMOGRAPHIC VARIABLES AND SPIRITUAL BELIEFS AND PRACTICES (N=23,826).

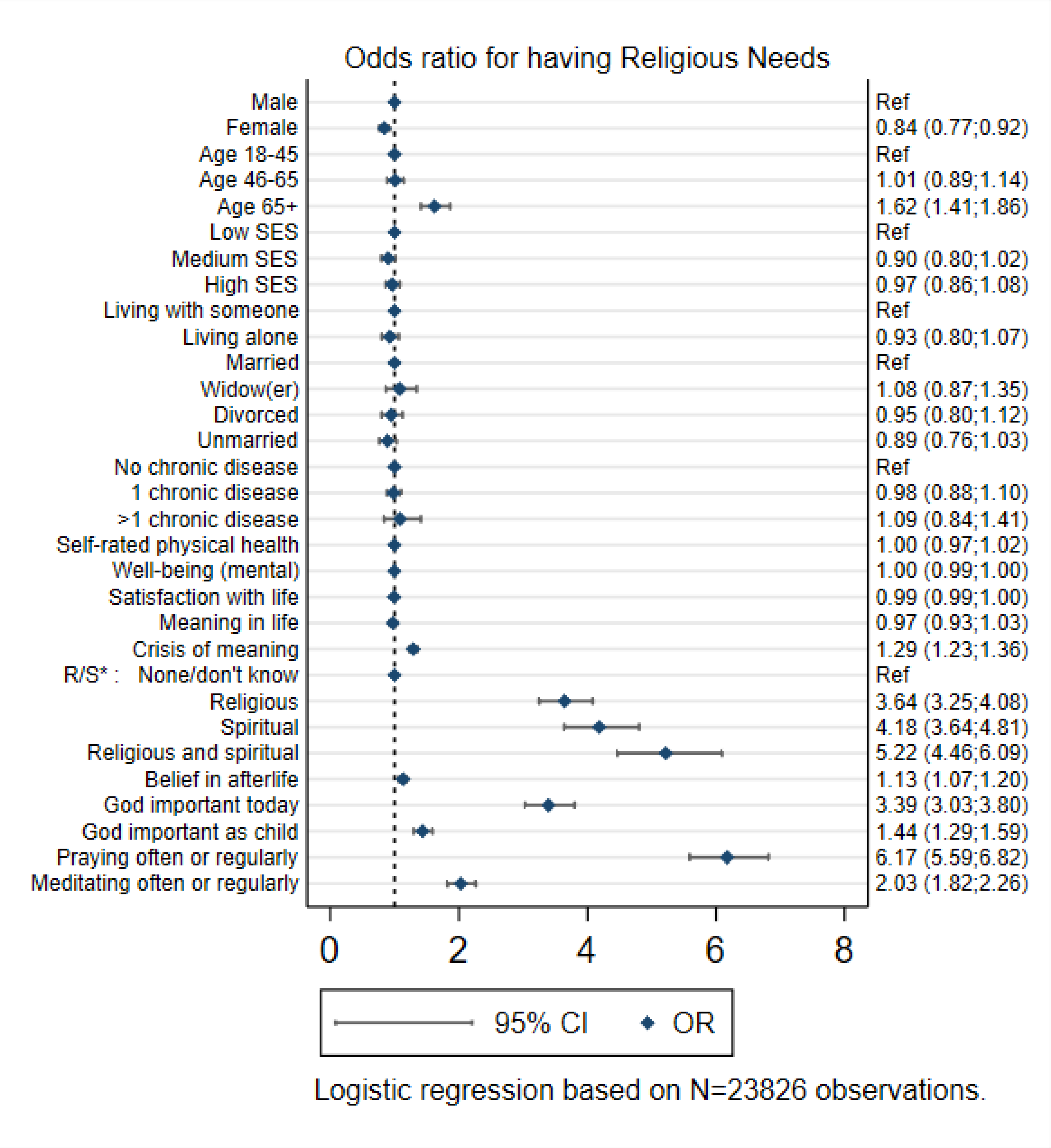

APPENDIX 4.2

ODDS RATIO FOR HAVING EXISTENTIAL NEEDS BY DEMOGRAPHIC VARIABLES AND SPIRITUAL BELIEFS AND PRACTICES (N=23,826).

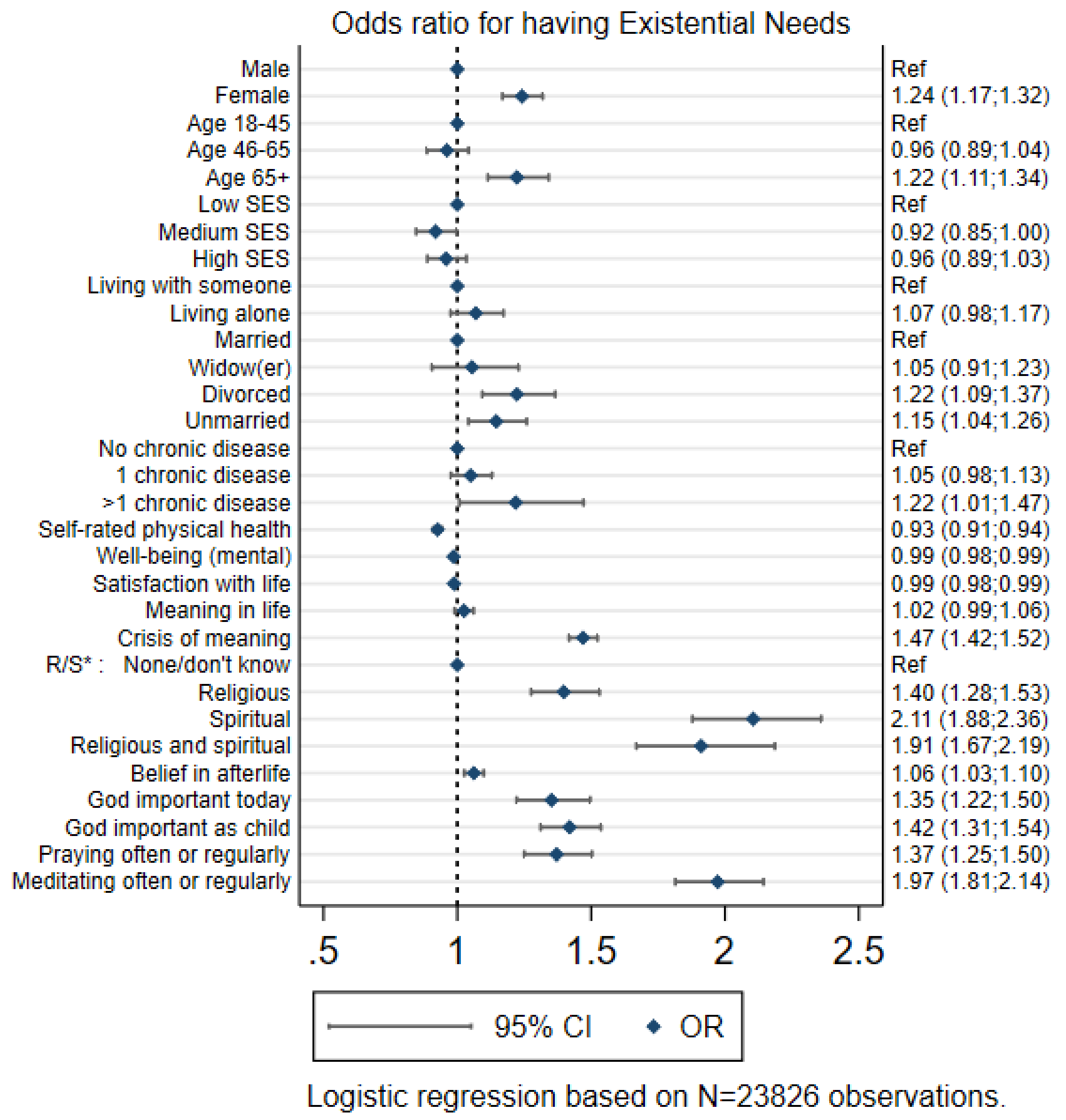

APPENDIX 4.3

ODDS RATIO FOR HAVING GENERATIVITY NEEDS BY DEMOGRAPHIC VARIABLES AND SPIRITUAL BELIEFS AND PRACTICES (N=23,826).

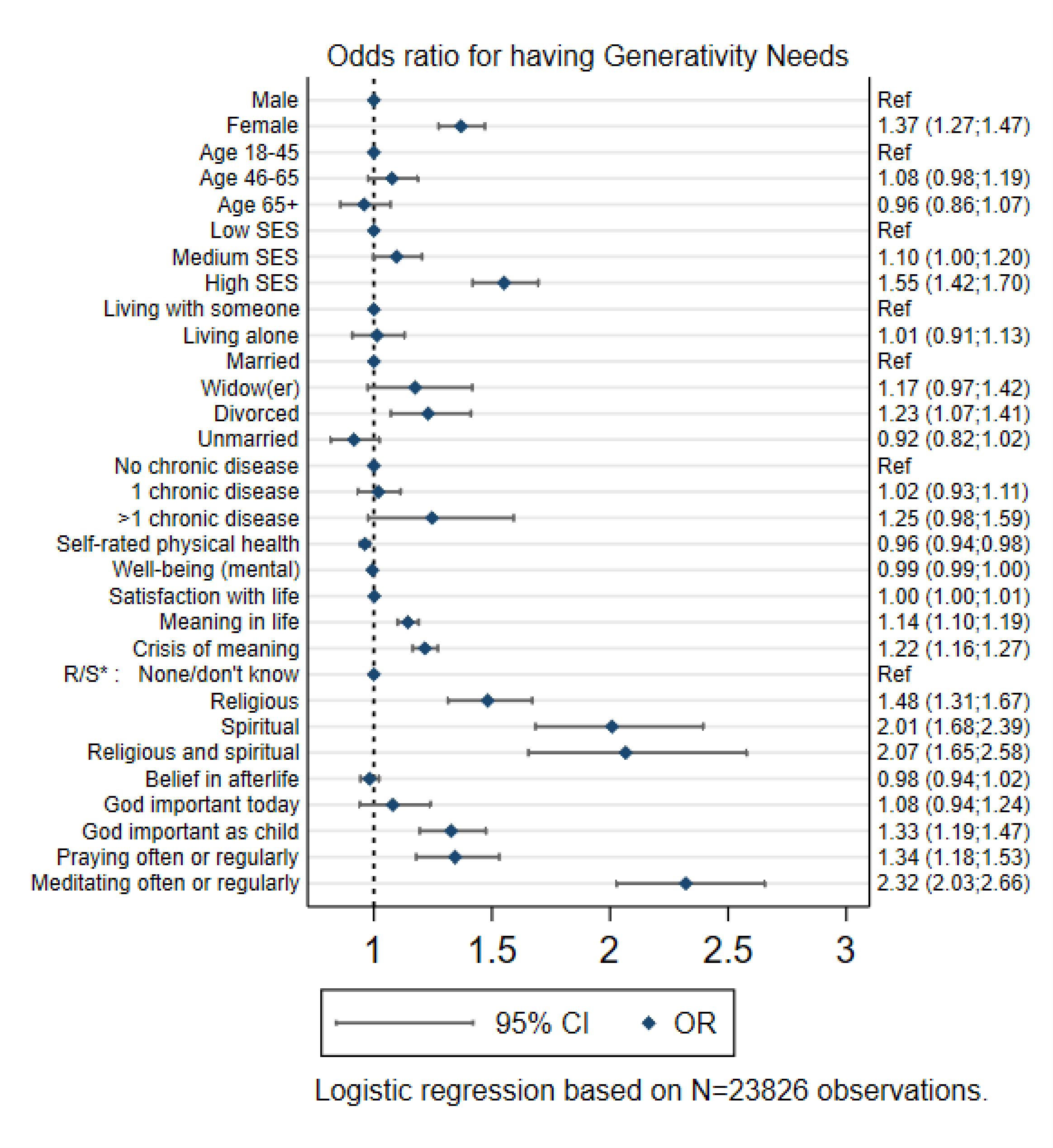

APPENDIX 4.4

ODDS RATIO FOR HAVING INNER PEACE NEEDS BY DEMOGRAPHIC VARIABLES AND SPIRITUAL BELIEFS AND PRACTICES (N=23,826).

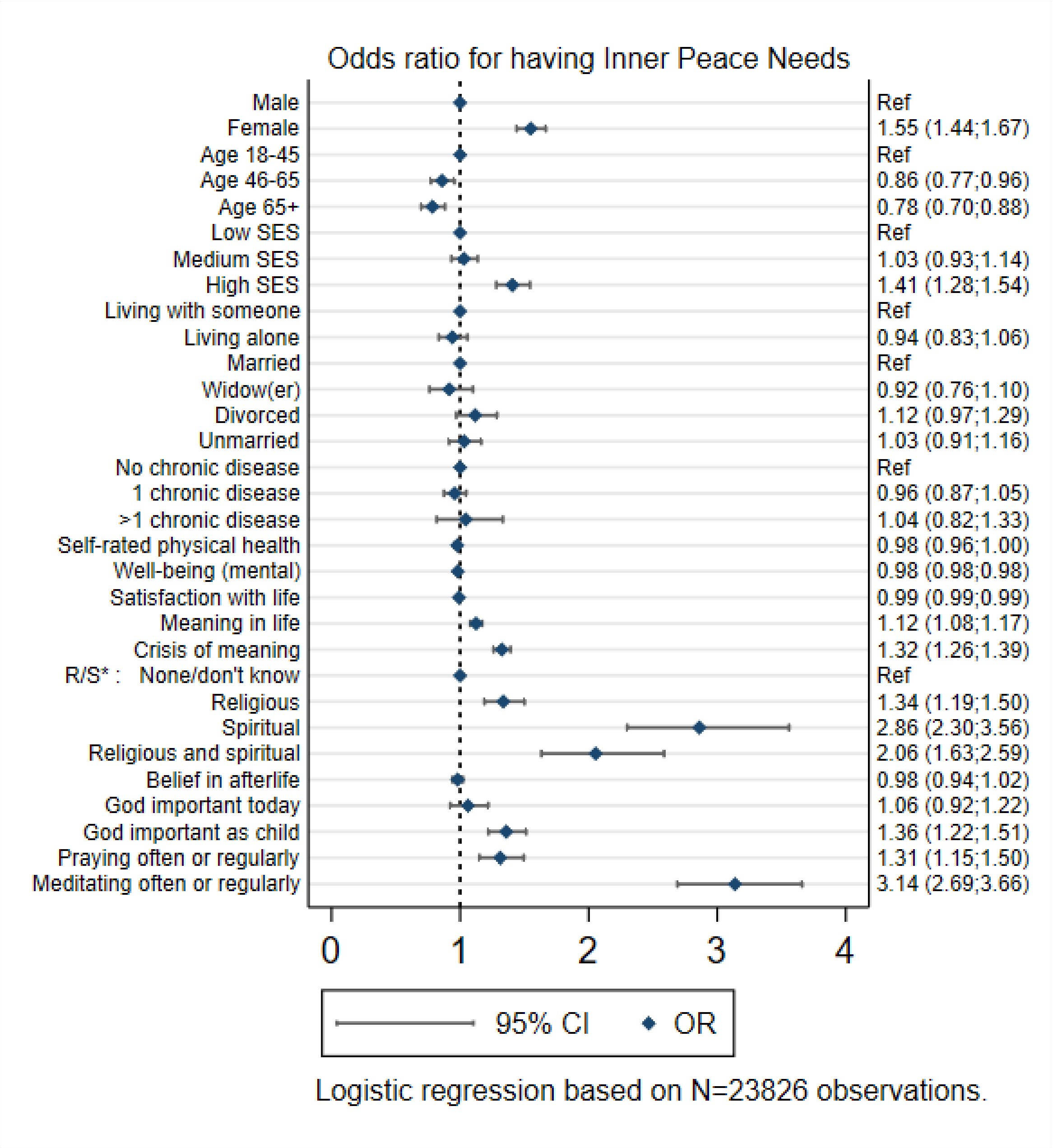

APPENDIX 5

UNI- AND MULTIVARIABLE LINEAR REGRESSION MODELS.

APPENDIX 5.1

ASSOCIATIONS BY LINEAR REGRESSION OF SPIRITUAL NEEDS OVERALL WITH DEMOGRAPHIC VARIABLES AND SPIRITUAL BELIEFS AND PRACTICES (N=23,826). MODEL 1 = SPIRITUAL NEEDS OVERALL AS DEPENDENT VARIABLE; DEMOGRAPHIC VARIABLES AS INDEPENDENT VARIABLES. MODEL 2 = SPIRITUAL NEEDS OVERALL AS DEPENDENT VARIABLE; DEMOGRAPHIC VARIABLES AND SURVEYED OUTCOMES AS INDEPENDENT VARIABLES.

	**UNIVARIATE REGRESSIONS**		**MODEL 1 (R^2=0.033)**			**MODEL 2 (R^2=0.357)**		
**VARIABLES**	**ESTIMATE**	**P-VAL**	**ESTIMATE**	**P-VAL**	**P-VAL**	**ESTIMATE**	**P-VAL**	**P-VAL**
**CONSTANT**			0.689 (0.659;0.719)	<0.001		0.656 (0.611;0.701)	<0.001	
**MALE**	Ref	Ref	Ref	Ref		Ref	Ref	
**FEMALE**	0.158 (0.146;0.171)	<0.001	0.154 (0.141;0.167)	<0.001		0.062 (0.051;0.073)	<0.001	
**AGE 18-45**	Ref	Ref	Ref	Ref	<0.001 †	Ref	Ref	<0.001 †
**AGE 46-65**	-0.025 (-0.041;-0.009)	0.003	-0.016 (-0.034;0.001)	0.072		0.004 (-0.010;0.019)	0.571	
**AGE 65+**	0.001 (-0.016;0.018)	0.918	0.018 (-0.002;0.038)	0.079		0.041 (0.024;0.058)	<0.001	
**LOW SES**	Ref	Ref	Ref	Ref	0.003†	Ref	Ref	<0.001 †
**MEDIUM SES**	-0.051 (-0.069;-0.032)	<0.001	-0.026 (-0.045;-0.008)	0.004		-0.007 (-0.022;0.008)	0.338	
**HIGH SES**	-0.060 (-0.076;-0.044)	<0.001	-0.027 (-0.044;-0.011)	0.001		0.019 (0.005;0.032)	0.009	
**LIVING ALONE**	Ref	Ref	Ref	Ref		Ref	Ref	
**LIVING WITH SOMEONE**	-0.094 (-0.108;-0.080)	<0.001	-0.078 (-0.098;-0.057)	<0.001		-0.009 (-0.025;0.008)	0.311	
**MARRIED**	Ref	Ref	Ref	Ref	<0.001 †	Ref	Ref	0.072†
**WIDOW(ER)**	0.070 (0.041;0.099)	<0.001	-0.051 (-0.085;-0.017)	0.003		-0.014 (-0.042;0.014)	0.335	
**DIVORCED**	0.099 (0.078;0.119)	<0.001	0.026 (0.001;0.050)	0.041		0.018 (-0.002;0.038)	0.083	
**UNMARRIED**	0.037 (0.021;0.053)	<0.001	-0.010 (-0.031;0.011)	0.353		-0.003 (-0.021;0.014)	0.707	
**NO CHRONIC DISEASE**	Ref	Ref	Ref	Ref	<0.001 †	Ref	Ref	0.290†
**1 CHRONIC DISEASE**	0.050 (0.034;0.066)	<0.001	0.036 (0.019;0.052)	<0.001		0.008 (-0.005;0.021)	0.239	
**>1 CHRONIC DISEASE**	0.125 (0.083;0.167)	<0.001	0.083 (0.042;0.125)	<0.001		0.020 (-0.014;0.054)	0.243	
**SELF-RATED PHYSICAL HEALTH**	-0.036 (-0.039;-0.033)	<0.001				-0.008 (-0.011;-0.004)	<0.001	
**WELL-BEING (MENTAL)**	-0.006 (-0.006;-0.005)	<0.001				-0.003 (-0.003;-0.002)	<0.001	
**SATISFACTION WITH LIFE**	-0.007 (-0.007;-0.006)	<0.001				-0.002 (-0.002;-0.001)	<0.001	
**MEANING IN LIFE**	-0.047 (-0.053;-0.042)	<0.001				0.025 (0.019;0.031)	<0.001	
**CRISIS OF MEANING**	0.106 (0.101;0.112)	<0.001				0.082 (0.075;0.088)	<0.001	
**R/S* : NONE/DON'T KNOW**	Ref	Ref				Ref	Ref	<0.001 †
**RELIGIOUS**	0.364 (0.348;0.380)	<0.001				0.121 (0.104;0.138)	<0.001	
**SPIRITUAL**	0.434 (0.412;0.456)	<0.001				0.183 (0.162;0.204)	<0.001	
**RELIGIOUS AND SPIRITUAL**	0.570 (0.545;0.594)	<0.001				0.186 (0.162;0.211)	<0.001	
**BELIEVE IN AFTERLIFE? - NO**	Ref	Ref				Ref	Ref	

**- YES**	0.427 (0.412;0.443)	<0.001				0.079 (0.063;0.096)	<0.001	
**- DON'T KNOW**	0.118 (0.104;0.132)	<0.001				-0.001 (-0.014;0.012)	0.876	
**GOD IMPORTANT TODAY? - NO**	Ref	Ref				Ref	Ref	
**- YES**	0.488 (0.473;0.503)	<0.001				0.154 (0.135;0.173)	<0.001	
**GOD IMPORTANT AS CHILD? - NO**	Ref	Ref				Ref	Ref	
**- YES**	0.349 (0.335;0.364)	<0.001				0.085 (0.071;0.100)	<0.001	
**DO YOU PRAY? - NO**	Ref	Ref				Ref	Ref	
**- YES**	0.523 (0.508;0.537)	<0.001				0.211 (0.194;0.228)	<0.001	
**DO YOU MEDITATE? - NO**	Ref	Ref				Ref	Ref	
**- YES**	0.450 (0.433;0.466)	<0.001				0.235 (0.220;0.250)	<0.001	

†Wald test estimates for categorical variables.

APPENDIX 5.2

ASSOCIATIONS BY LINEAR REGRESSION OF RELIGIOUS NEEDS WITH DEMOGRAPHIC VARIABLES AND SPIRITUAL BELIEFS AND PRACTICES (N=23,826). MODEL 1 = RELIGIOUS NEEDS AS DEPENDENT VARIABLE; DEMOGRAPHIC VARIABLES AS INDEPENDENT VARIABLES. MODEL 2 = RELIGIOUS NEEDS AS DEPENDENT VARIABLE; DEMOGRAPHIC VARIABLES AND SURVEYED OUTCOMES AS INDEPENDENT VARIABLES.

	UNIVARIATE REGRESSIONS		MODEL 1 (R^2=0.022)			MODEL 2 (R^2=0.512)		
**VARIABLES**	**ESTIMATE**	**P-VAL**	**ESTIMATE**	**P-VAL**	**P-VAL**	**ESTIMATE**	**P-VAL**	**P-VAL**
**CONSTANT**			0.278 (0.244;0.312)	<0.001		0.065 (0.021;0.109)	0.004	
**MALE**	Ref	Ref	Ref	Ref		Ref	Ref	
**FEMALE**	0.088 (0.074;0.103)	<0.001s	0.096 (0.081;0.111)	<0.001		-0.032 (-0.043;-0.021)	<0.001	
**AGE 18-45**	Ref	Ref	Ref	Ref/	<0.001†	Ref	Ref	<0.001†
**AGE 46-65**	0.026 (0.008;0.044)	0.005	0.016 (-0.004;0.036)	0.112		-0.012 (-0.027;0.002)	0.090	
**AGE 65+**	0.144 (0.124;0.163)	<0.001	0.132 (0.109;0.155)	<0.001		0.045 (0.028;0.062)	<0.001	
**LOW SES**	Ref	Ref	Ref	Ref	0.002†	Ref	Ref	0.215†
**MEDIUM SES**	-0.037 (-0.057;-0.016)	<0.001	-0.022 (-0.043;-0.001)	0.037		-0.013 (-0.027;0.002)	0.089	
**HIGH SES**	-0.048 (-0.067;-0.030)	<0.001	-0.034 (-0.053;-0.015)	<0.001		-0.010 (-0.023;0.004)	0.163	
**LIVING ALONE**	Ref	Ref	Ref	Ref		Ref	Ref	
**LIVING WITH SOMEONE**	-0.052 (-0.068;-0.036)	<0.001	-0.076 (-0.099;-0.053)	<0.001		-0.016 (-0.032;0.001)	0.058	
**MARRIED**	Ref	Ref	Ref	Ref	<0.001†	Ref	Ref	0.126†
**WIDOW(ER)**	0.108 (0.075;0.141)	<0.001	-0.039 (-0.078;-0.000)	0.049		-0.012 (-0.039;0.015)	0.390	
**DIVORCED**	0.024 (0.002;0.047)	0.035	-0.038 (-0.066;-0.010)	0.008		-0.024 (-0.044;-0.004)	0.018	
**UNMARRIED**	-0.060 (-0.077;-0.042)	<0.001	-0.066 (-0.089;-0.042)	<0.001		-0.012 (-0.029;0.005)	0.173	
**NO CHRONIC DISEASE**	Ref	Ref	Ref	Ref	0.832†	Ref	Ref	0.239†
**1 CHRONIC DISEASE**	0.030 (0.012;0.048)	0.001	0.003 (-0.016;0.021)	0.770		-0.010 (-0.024;0.003)	0.117	
**>1 CHRONIC DISEASE**	0.083 (0.036;0.130)	<0.001	0.013 (-0.034;0.061)	0.578		-0.014 (-0.047;0.020)	0.418	
**SELF-RATED PHYSICAL HEALTH**	-0.005 (-0.009;-0.002)	0.003				-0.002 (-0.005;0.001)	0.184	
**WELL-BEING (MENTAL)**	-0.001 (-0.001;-0.000)	0.006				-0.000 (-0.001;-0.000)	0.040	
**SATISFACTION WITH LIFE**	-0.001 (-0.001;-0.000)	0.003				-0.000 (-0.001;0.000)	0.163	
**MEANING IN LIFE**	0.009 (0.003;0.016)	0.005				0.005 (-0.001;0.011)	0.117	
**CRISIS OF MEANING**	0.025 (0.019;0.032)	<0.001				0.027 (0.021;0.034)	<0.001	
**R/S* : NONE/DON'T KNOW**	Ref	Ref				Ref	Ref	<0.001†
**RELIGIOUS**	0.671 (0.654;0.688)	<0.001				0.188 (0.171;0.205)	<0.001	
**SPIRITUAL**	0.377 (0.354;0.400)	<0.001				0.076 (0.055;0.096)	<0.001	
**RELIGIOUS AND SPIRITUAL**	0.764 (0.738;0.790)	<0.001				0.166 (0.141;0.190)	<0.001	
**BELIEVE IN AFTERLIFE? - NO**	Ref	Ref				Ref	Ref	
**- YES**	0.633 (0.616;0.650)	<0.001				0.153 (0.137;0.169)	<0.001	
**- DON'T KNOW**	0.126 (0.111;0.142)	<0.001				-0.018 (-0.031;-0.006)	0.004	

**GOD IMPORTANT TODAY? - NO**	Ref	Ref				Ref	Ref	
**- YES**	0.858 (0.843;0.872)	<0.001				0.348 (0.329;0.366)	<0.001	
**GOD IMPORTANT AS CHILD? - NO**	Ref	Ref				Ref	Ref	
**- YES**	0.536 (0.521;0.552)	<0.001				0.070 (0.055;0.084)	<0.001	
**DO YOU PRAY? - NO**	Ref	Ref				Ref	Ref	
**- YES**	0.878 (0.864;0.892)	<0.001				0.444 (0.427;0.460)	<0.001	
**DO YOU MEDITATE? - NO**	Ref	Ref				Ref	Ref	
**- YES**	0.460 (0.442;0.479)	<0.001				0.176 (0.161;0.191)	<0.001	

† = Wald test estimate for categorical variables.

APPENDIX 5.3

ASSOCIATIONS BY LINEAR REGRESSION OF EXISTENTIAL NEEDS WITH DEMOGRAPHIC VARIABLES AND SPIRITUAL BELIEFS AND PRACTICES (N=23,826). MODEL 1 = EXISTENTIAL NEEDS AS DEPENDENT VARIABLE; DEMOGRAPHIC VARIABLES AS INDEPENDENT VARIABLES. MODEL 2 = EXISTENTIAL NEEDS AS DEPENDENT VARIABLE; DEMOGRAPHIC VARIABLES AND SURVEYED OUTCOMES AS INDEPENDENT VARIABLES.

	**UNIVARIATE REGRESSIONS**		**MODEL 1 (R^2=0.033)**			**MODEL 2 (R^2=0.270)**		
**VARIABLES**	**ESTIMATE**	**P-VAL**	**ESTIMATE**	**P-VAL**	**P-VAL**	**ESTIMATE**	**P-VAL**	**P-VAL**
**CONSTANT**			0.589 (0.555;0.623)	<0.001		0.747 (0.693;0.801)	<0.001	
**MALE**	Ref	Ref	Ref	Ref		Ref	Ref	
**FEMALE**	0.123 (0.108;0.137)	<0.001	0.112 (0.098;0.127)	<0.001		0.046 (0.033;0.059)	<0.001	
**AGE 18-45**	Ref	Ref	Ref	Ref	<0.001 †	Ref	Ref	<0.001 †
**AGE 46-65**	-0.069 (-0.087;-0.051)	<0.001	-0.045 (-0.065;-0.025)	<0.001		0.002 (-0.015;0.020)	0.792	
**AGE 65+**	-0.035 (-0.054;-0.015)	<0.001	-0.004 (-0.027;0.019)	0.736		0.071 (0.051;0.092)	<0.001	
**LOW SES**	Ref	Ref	Ref	Ref	<0.001 †	Ref	Ref	0.013†
**MEDIUM SES**	-0.084 (-0.105;-0.063)	<0.001	-0.055 (-0.076;-0.035)	<0.001		-0.019 (-0.037;-0.001)	0.043	
**HIGH SES**	-0.148 (-0.166;-0.130)	<0.001	-0.103 (-0.122;-0.084)	<0.001		-0.025 (-0.042;-0.008)	0.003	
**LIVING ALONE**	Ref	Ref	Ref	Ref		Ref	Ref	
**LIVING WITH SOMEONE**	-0.135 (-0.152;-0.119)	<0.001	-0.077 (-0.100;-0.054)	<0.001		0.001 (-0.019;0.021)	0.906	
**MARRIED**	Ref	Ref	Ref	Ref	<0.001 †	Ref	Ref	0.005†
**WIDOW(ER)**	0.082 (0.050;0.115)	<0.001	-0.046 (-0.085;-0.007)	0.020		-0.009 (-0.043;0.025)	0.607	
**DIVORCED**	0.141 (0.119;0.164)	<0.001	0.059 (0.031;0.087)	<0.001		0.038 (0.014;0.063)	0.002	
**UNMARRIED**	0.107 (0.090;0.125)	<0.001	0.037 (0.014;0.061)	0.002		0.012 (-0.009;0.033)	0.258	
**NO CHRONIC DISEASE**	Ref	Ref	Ref	Ref	<0.001 †	Ref	Ref	0.023†
**1 CHRONIC DISEASE**	0.073 (0.054;0.091)	<0.001	0.057 (0.039;0.076)	<0.001		0.016 (0.000;0.032)	0.048	
**>1 CHRONIC DISEASE**	0.185 (0.138;0.232)	<0.001	0.143 (0.096;0.190)	<0.001		0.044 (0.003;0.086)	0.035	
**SELF-RATED PHYSICAL HEALTH**	-0.062 (-0.066;-0.059)	<0.001				-0.015 (-0.019;-0.011)	<0.001	
**WELL-BEING (MENTAL)**	-0.009 (-0.009;-0.008)	<0.001				-0.003 (-0.004;-0.003)	<0.001	
**SATISFACTION WITH LIFE**	-0.011 (-0.012;-0.011)	<0.001				-0.003 (-0.004;-0.002)	<0.001	
**MEANING IN LIFE**	-0.111 (-0.117;-0.104)	<0.001				0.013 (0.005;0.020)	0.001	
**CRISIS OF MEANING**	0.174 (0.168;0.181)	<0.001				0.118 (0.111;0.126)	<0.001	
**R/S* : NONE/DON'T KNOW**	Ref	Ref				Ref	Ref	<0.001 †
**RELIGIOUS**	0.208 (0.189;0.227)	<0.001				0.060 (0.039;0.080)	<0.001	
**SPIRITUAL**	0.394 (0.368;0.420)	<0.001				0.186 (0.160;0.211)	<0.001	
**RELIGIOUS AND SPIRITUAL**	0.435 (0.405;0.464)	<0.001				0.160 (0.130;0.190)	<0.001	
**BELIEVE IN AFTERLIFE? - NO**	Ref	Ref				Ref	Ref	
**- YES**	0.332 (0.314;0.350)	<0.001				0.081 (0.061;0.101)	<0.001	

**- DON'T KNOW**	0.120 (0.103;0.137)	<0.001				0.030 (0.015;0.045)	<0.001	
**GOD IMPORTANT TODAY? - NO**	Ref	Ref				Ref	Ref	
**- YES**	0.326 (0.308;0.344)	<0.001				0.096 (0.073;0.119)	<0.001	
**GOD IMPORTANT AS CHILD? - NO**	Ref	Ref				Ref	Ref	
**- YES**	0.256 (0.239;0.273)	<0.001				0.080 (0.062;0.097)	<0.001	
**DO YOU PRAY? - NO**	Ref	Ref				Ref	Ref	
**- YES**	0.354 (0.336;0.371)	<0.001				0.117 (0.096;0.138)	<0.001	
**DO YOU MEDITATE? - NO**	Ref	Ref				Ref	Ref	
**- YES**	0.364 (0.345;0.383)	<0.001				0.193 (0.174;0.211)	<0.001	

† = Wald test estimate for categorical variables.

APPENDIX 5.4

ASSOCIATIONS BY LINEAR REGRESSION OF GENERATIVITY NEEDS WITH DEMOGRAPHIC VARIABLES AND SPIRITUAL BELIEFS AND PRACTICES (N=23,826). MODEL 1 = GENERATIVITY NEEDS AS DEPENDENT VARIABLE; DEMOGRAPHIC VARIABLES AS INDEPENDENT VARIABLES. MODEL 2 = GENERATIVITY NEEDS AS DEPENDENT VARIABLE; DEMOGRAPHIC VARIABLES AND SURVEYED OUTCOMES AS INDEPENDENT VARIABLES.

	**UNIVARIATE REGRESSIONS**		**MODEL 1 (R^2=0.020)**			**MODEL 2 (R^2=0.124)**		
**VARIABLES**	**ESTIMATE**	**P-VAL**	**ESTIMATE**	**P-VAL**	**P-VAL**	**ESTIMATE**	**P-VAL**	**P-VAL**
**CONSTANT**			0.965 (0.923;1.007)	<0.001		0.648 (0.575;0.721)	<0.001	
**MALE**	Ref	Ref	Ref	Ref		Ref	Ref	
**FEMALE**	0.162 (0.144;0.180)	<0.001	0.161 (0.143;0.179)	<0.001		0.083 (0.065;0.101)	<0.001	
**AGE 18-45**	Ref	Ref	Ref	Ref	<0.001 †	Ref	Ref	<0.001 †
**AGE 46-65**	0.052 (0.029;0.074)	<0.001	0.029 (0.004;0.054)	0.021		0.036 (0.012;0.060)	0.004	
**AGE 65+**	0.083 (0.059;0.107)	<0.001	0.061 (0.033;0.090)	<0.001		0.074 (0.047;0.102)	<0.001	
**LOW SES**	Ref	Ref	Ref	Ref		Ref	Ref	
**MEDIUM SES**	-0.021 (-0.046;0.005)	0.109	-0.003 (-0.029;0.022)	0.798	<0.001 †	-0.003 (-0.027;0.021)	0.815	<0.001 †
**HIGH SES**	0.027 (0.005;0.050)	0.018	0.046 (0.023;0.069)	<0.001		0.057 (0.034;0.079)	<0.001	
**LIVING ALONE**	Ref	Ref	Ref	Ref		Ref	Ref	
**LIVING WITH SOMEONE**	-0.042 (-0.062;-0.022)	<0.001	-0.042 (-0.070;-0.013)	0.004		0.005 (-0.022;0.032)	0.720	
**MARRIED**	Ref	Ref	Ref	Ref	<0.001 †	Ref	Ref	<0.001 †
**WIDOW(ER)**	0.090 (0.049;0.130)	<0.001	0.005 (-0.043;0.053)	0.852		0.036 (-0.010;0.081)	0.125	
**DIVORCED**	0.089 (0.061;0.117)	<0.001	0.053 (0.019;0.088)	0.003		0.051 (0.018;0.084)	0.002	
**UNMARRIED**	-0.065 (-0.087;-0.043)	<0.001	-0.058 (-0.088;-0.029)	<0.001		-0.039 (-0.067;-0.011)	0.007	
**NO CHRONIC DISEASE**	Ref	Ref	Ref	Ref	<0.001 †	Ref	Ref	0.019†
**1 CHRONIC DISEASE**	0.057 (0.034;0.080)	<0.001	0.042 (0.019;0.065)	<0.001		0.026 (0.004;0.048)	0.019	
**>1 CHRONIC DISEASE**	0.130 (0.071;0.188)	<0.001	0.084 (0.025;0.142)	0.005		0.051 (-0.004;0.107)	0.071	
**SELF-RATED PHYSICAL HEALTH**	-0.019 (-0.023;-0.014)	<0.001				-0.012 (-0.018;-0.007)	<0.001	
**WELL-BEING (MENTAL)**	-0.002 (-0.003;-0.002)	<0.001				-0.002 (-0.002;-0.001)	<0.001	
**SATISFACTION WITH LIFE**	-0.002 (-0.002;-0.001)	<0.001				0.000 (-0.000;0.001)	0.286	
**MEANING IN LIFE**	0.015 (0.007;0.023)	<0.001				0.057 (0.047;0.067)	<0.001	
**CRISIS OF MEANING**	0.050 (0.042;0.058)	<0.001				0.077 (0.067;0.088)	<0.001	
**R/S* : NONE/DON'T KNOW**	Ref	Ref				Ref	Ref	<0.001 †
**RELIGIOUS**	0.320 (0.297;0.344)	<0.001				0.134 (0.106;0.162)	<0.001	
**SPIRITUAL**	0.407 (0.375;0.439)	<0.001				0.213 (0.179;0.248)	<0.001	
**RELIGIOUS AND SPIRITUAL**	0.491 (0.455;0.528)	<0.001				0.200 (0.159;0.240)	<0.001	
**BELIEVE IN AFTERLIFE? - NO**	Ref	Ref				Ref	Ref	
**- YES**	0.346 (0.324;0.369)	<0.001				0.064 (0.037;0.090)	<0.001	
**- DON'T KNOW**	0.087 (0.066;0.107)	<0.001				-0.008 (-0.028;0.013)	0.459	

**GOD IMPORTANT TODAY? - NO**	Ref	Ref				Ref	Ref	
**- YES**	0.379 (0.357;0.401)	<0.001				0.072 (0.041;0.103)	<0.001	
**GOD IMPORTANT AS CHILD? - NO**	Ref	Ref				Ref	Ref	
**- YES**	0.309 (0.288;0.329)	<0.001				0.113 (0.089;0.137)	<0.001	
**DO YOU PRAY? - NO**	Ref	Ref				Ref	Ref	
**- YES**	0.395 (0.373;0.416)	<0.001				0.096 (0.068;0.124)	<0.001	
**DO YOU MEDITATE? - NO**	Ref	Ref				Ref	Ref	
**- YES**	0.407 (0.383;0.430)	<0.001				0.234 (0.209;0.259)	<0.001	

† = Wald test estimate for categorical variables.

APPENDIX 5.5

ASSOCIATIONS BY LINEAR REGRESSION OF INNER PEACE NEEDS WITH DEMOGRAPHIC VARIABLES AND SPIRITUAL BELIEFS AND PRACTICES (N=23,826). MODEL 1 = INNER PEACE NEEDS AS DEPENDENT VARIABLE; DEMOGRAPHIC VARIABLES AS INDEPENDENT VARIABLES. MODEL 2 = INNER PEACE NEEDS AS DEPENDENT VARIABLE; DEMOGRAPHIC VARIABLES AND SURVEYED OUTCOMES AS INDEPENDENT VARIABLES.

	UNIVARIATE REGRESSIONS		MODEL 1 (R^2=0.056)			MODEL 2 (R^2=0.244)		
**VARIABLES**	**ESTIMATE**	**P-VAL**	**ESTIMATE**	**P-VAL**	**P-VAL**	**ESTIMATE**	**P-VAL**	**P-VAL**
**CONSTANT**			1.181 (1.135;1.227)	<0.001		1.412 (1.337;1.488)	<0.001	
**MALE**	Ref	Ref	Ref	Ref		Ref	Ref	
**FEMALE**	0.314 (0.294;0.333)	<0.001	0.295 (0.275;0.315)	<0.001		0.204 (0.185;0.222)	<0.001	
**AGE 18-45**	Ref	Ref	Ref	Ref	<0.001 †	Ref	Ref	<0.001 †
**AGE 46-65**	-0.111 (-0.136;-0.086)	<0.001	-0.068 (-0.095;-0.041)	<0.001		0.001 (-0.024;0.025)	0.956	
**AGE 65+**	-0.241 (-0.268;-0.215)	<0.001	-0.164 (-0.194;-0.133)	<0.001		-0.042 (-0.071;-0.014)	0.004	
**LOW SES**	Ref	Ref	Ref	Ref	0.007†	Ref	Ref	<0.001 †
**MEDIUM SES**	-0.051 (-0.079;-0.022)	<0.001	-0.013 (-0.041;0.015)	0.363		0.013 (-0.012;0.038)	0.294	
**HIGH SES**	-0.034 (-0.059;-0.009)	0.008	0.024 (-0.002;0.049)	0.068		0.088 (0.065;0.111)	<0.001	
**LIVING ALONE**	Ref	Ref	Ref	Ref		Ref	Ref	
**LIVING WITH SOMEONE**	-0.146 (-0.169;-0.124)	<0.001	-0.118 (-0.149;-0.087)	<0.001		-0.026 (-0.054;0.002)	0.069	
**MARRIED**	Ref	Ref	Ref	Ref	<0.001 †	Ref	Ref	<0.001 †
**WIDOW(ER)**	-0.026 (-0.070;0.019)	0.263	-0.132 (-0.184;-0.080)	<0.001		-0.073 (-0.120;-0.026)	0.002	
**DIVORCED**	0.154 (0.123;0.186)	<0.001	0.043 (0.005;0.081)	0.026		0.016 (-0.018;0.050)	0.347	
**UNMARRIED**	0.178 (0.154;0.203)	<0.001	0.051 (0.019;0.083)	0.002		0.022 (-0.007;0.051)	0.137	
**NO CHRONIC DISEASE**	Ref	Ref	Ref	Ref	<0.001 †	Ref	Ref	0.890†
**1 CHRONIC DISEASE**	0.038 (0.013;0.063)	0.003	0.047 (0.022;0.071)	<0.001		0.005 (-0.017;0.028)	0.637	
**>1 CHRONIC DISEASE**	0.094 (0.029;0.159)	0.005	0.099 (0.036;0.163)	0.002		0.004 (-0.053;0.062)	0.879	
**SELF-RATED PHYSICAL HEALTH**	-0.061 (-0.066;-0.057)	<0.001				0.000 (-0.005;0.005)	0.934	
**WELL-BEING (MENTAL)**	-0.012 (-0.013;-0.012)	<0.001				-0.007 (-0.008;-0.006)	<0.001	
**SATISFACTION WITH LIFE**	-0.013 (-0.014;-0.013)	<0.001				-0.004 (-0.005;-0.003)	<0.001	
**MEANING IN LIFE**	-0.100 (-0.109;-0.091)	<0.001				0.042 (0.031;0.052)	<0.001	
**CRISIS OF MEANING**	0.182 (0.174;0.191)	<0.001				0.113 (0.103;0.124)	<0.001	
**R/S* : NONE/DON'T KNOW**	Ref	Ref				Ref	Ref	<0.001 †
**RELIGIOUS**	0.181 (0.155;0.207)	<0.001				0.097 (0.068;0.126)	<0.001	
**SPIRITUAL**	0.607 (0.571;0.642)	<0.001				0.308 (0.273;0.343)	<0.001	
**RELIGIOUS AND SPIRITUAL**	0.560 (0.520;0.600)	<0.001				0.243 (0.201;0.284)	<0.001	
**BELIEVE IN AFTERLIFE? - NO**	Ref	Ref				Ref	Ref	
**- YES**	0.342 (0.317;0.367)	<0.001				-0.019 (-0.046;0.009)	0.179	

**- DON'T KNOW**	0.133 (0.110;0.156)	<0.001				-0.015 (-0.036;0.006)	0.169	
**GOD IMPORTANT TODAY? - NO**	Ref	Ref				Ref	Ref	
**- YES**	0.284 (0.260;0.309)	<0.001				0.032 (0.000;0.063)	0.050	
**GOD IMPORTANT AS CHILD? - NO**	Ref	Ref				Ref	Ref	
**- YES**	0.250 (0.226;0.273)	<0.001				0.088 (0.064;0.113)	<0.001	
**DO YOU PRAY? - NO**	Ref	Ref				Ref	Ref	
**- YES**	0.372 (0.348;0.396)	<0.001				0.118 (0.089;0.147)	<0.001	
**DO YOU MEDITATE? - NO**	Ref	Ref				Ref	Ref	
**- YES**	0.604 (0.579;0.630)	<0.001				0.387 (0.362;0.413)	<0.001	

† = Wald test estimate for categorical variables.

APPENDIX 6

FOREST PLOTS OF MULTIVARIABLE LINEAR REGRESSION MODELS.

APPENDIX 6.1

FOREST PLOT OF ASSOCIATIONS BY LINEAR REGRESSION OF SPIRITUAL NEEDS OVERALL WITH DEMOGRAPHIC VARIABLES AND SPIRITUAL BELIEFS AND PRACTICES (N=23,826).

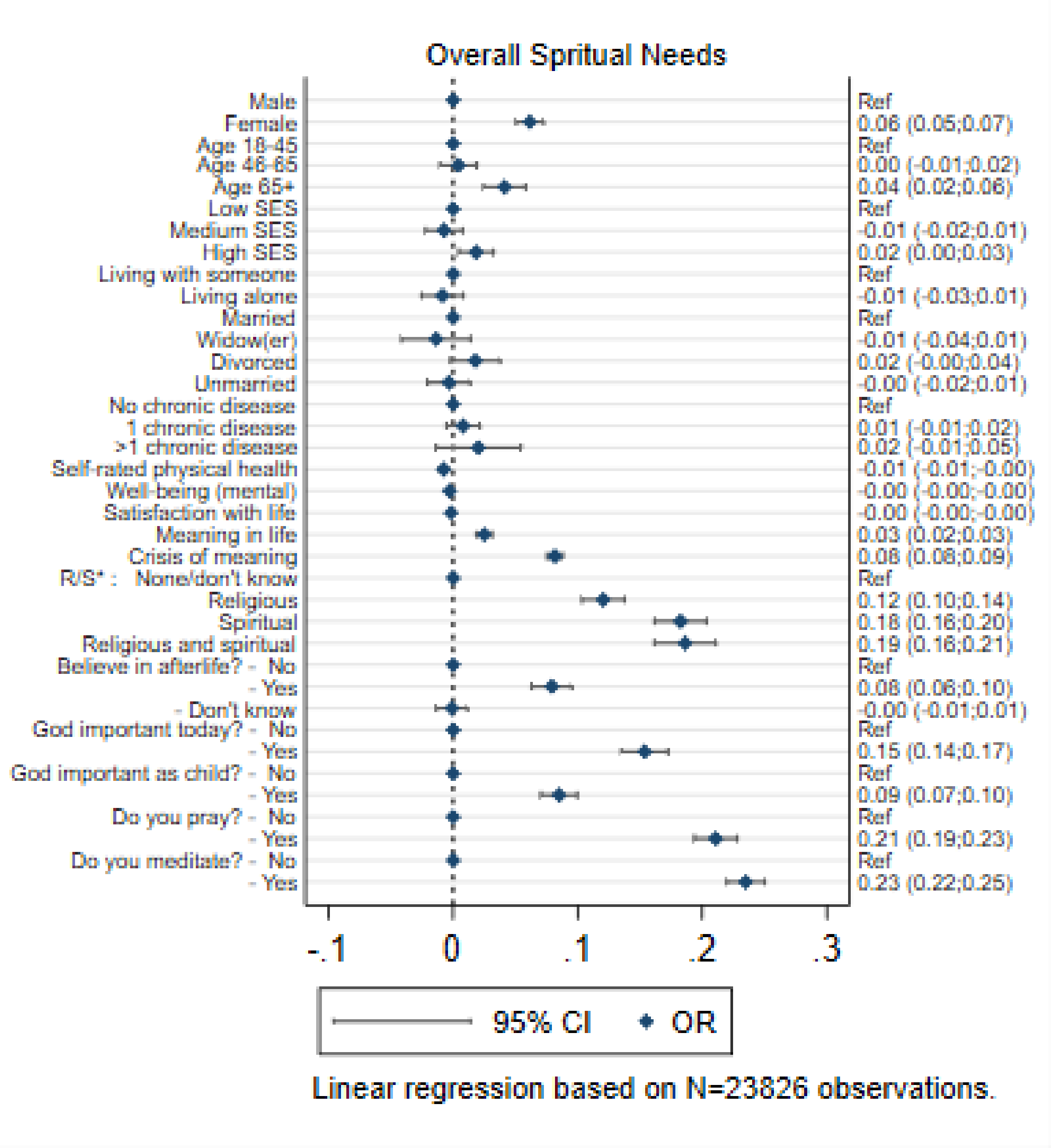

APPENDIX 6.2

FOREST PLOT OF ASSOCIATIONS BY LINEAR REGRESSION OF RELIGIOUS NEEDS WITH DEMOGRAPHIC VARIABLES AND SPIRITUAL BELIEFS AND PRACTICES (N=23,826).

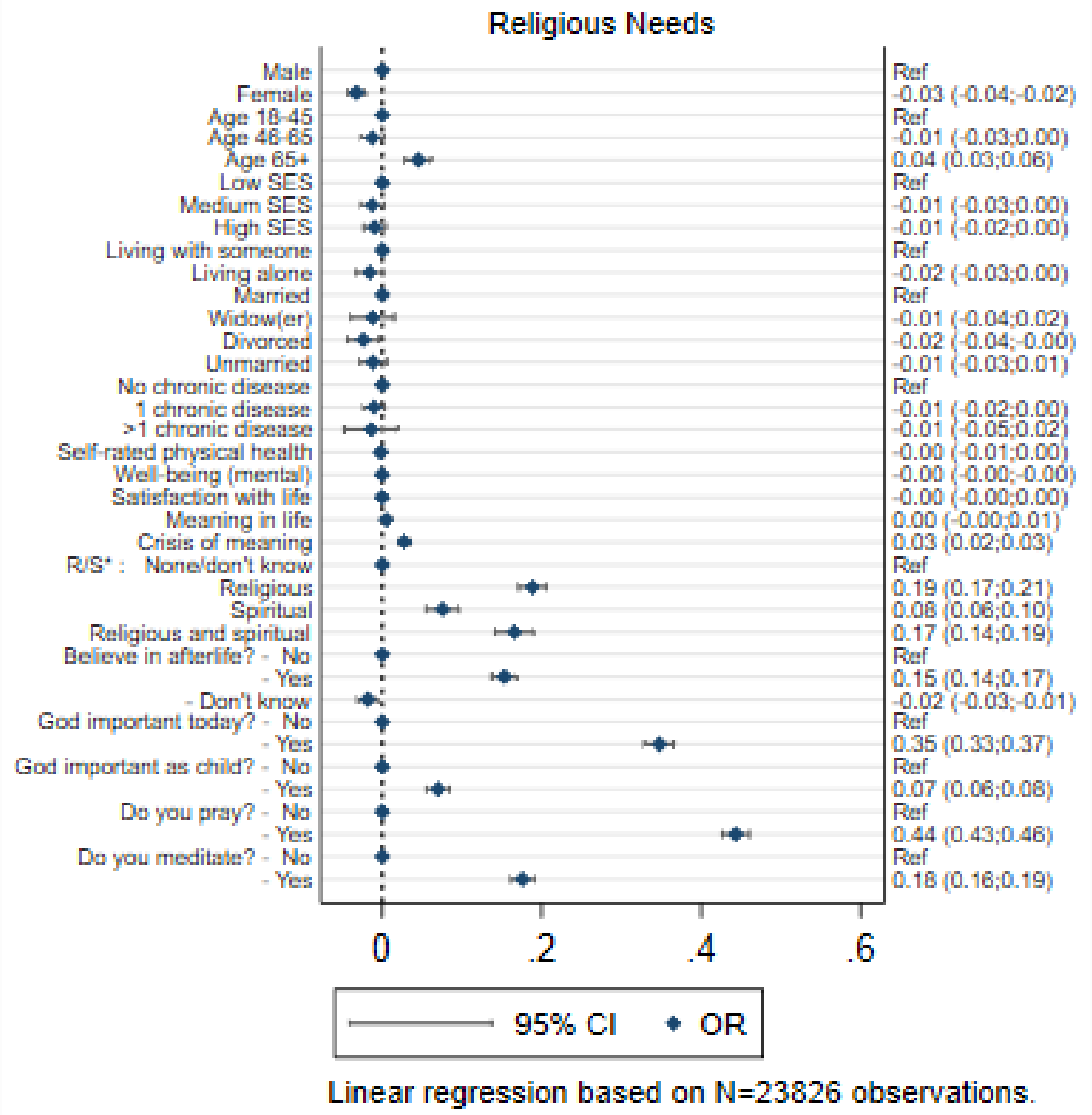

APPENDIX 6.3

FOREST PLOT OF ASSOCIATIONS BY LINEAR REGRESSION OF EXISTENTIAL NEEDS WITH DEMOGRAPHIC VARIABLES AND SPIRITUAL BELIEFS AND PRACTICES (N=23,826).

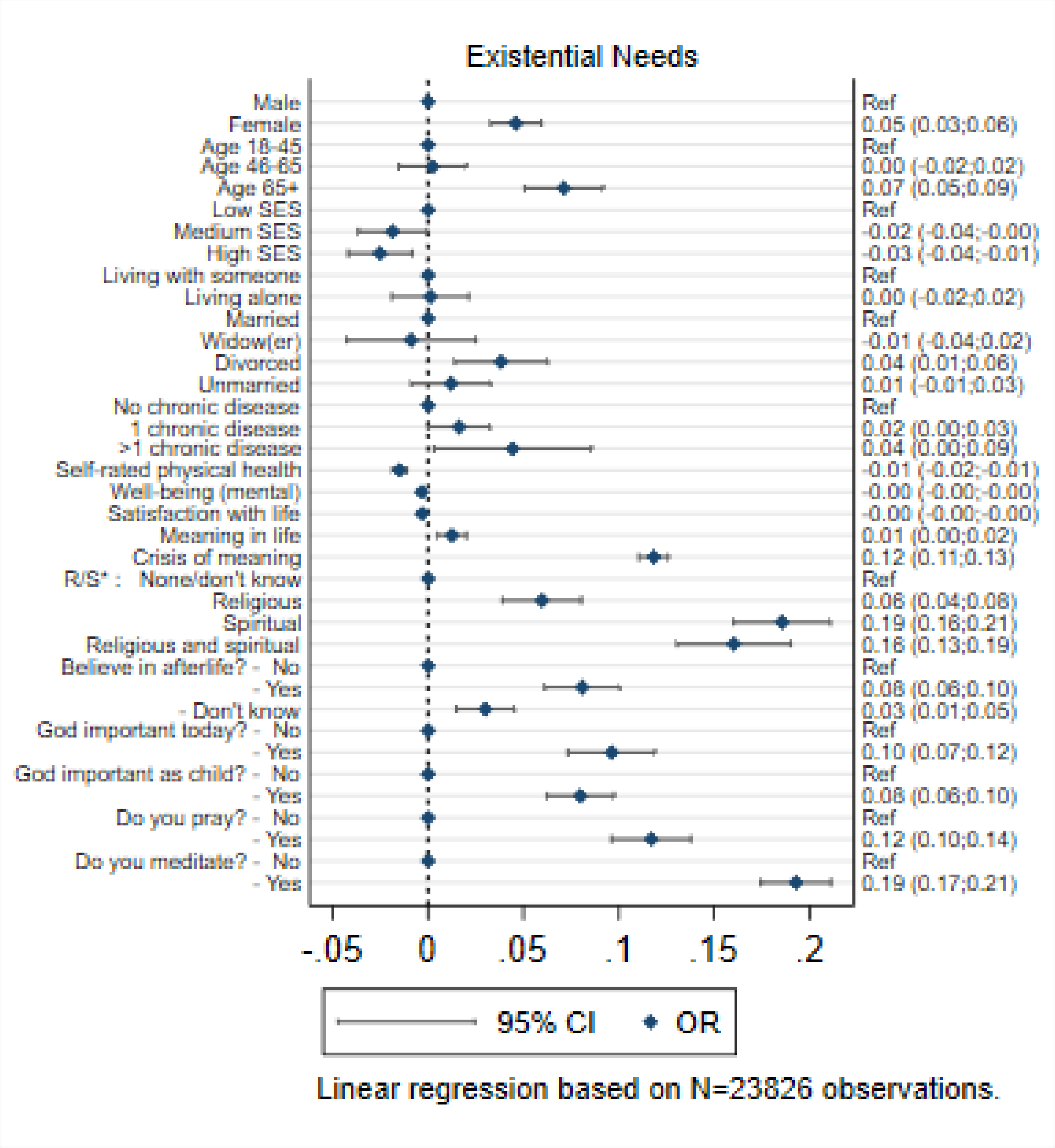

APPENDIX 6.4

FOREST PLOT OF ASSOCIATIONS BY LINEAR REGRESSION OF GENERATIVITY NEEDS WITH DEMOGRAPHIC VARIABLES AND SPIRITUAL BELIEFS AND PRACTICES (N=23,826).

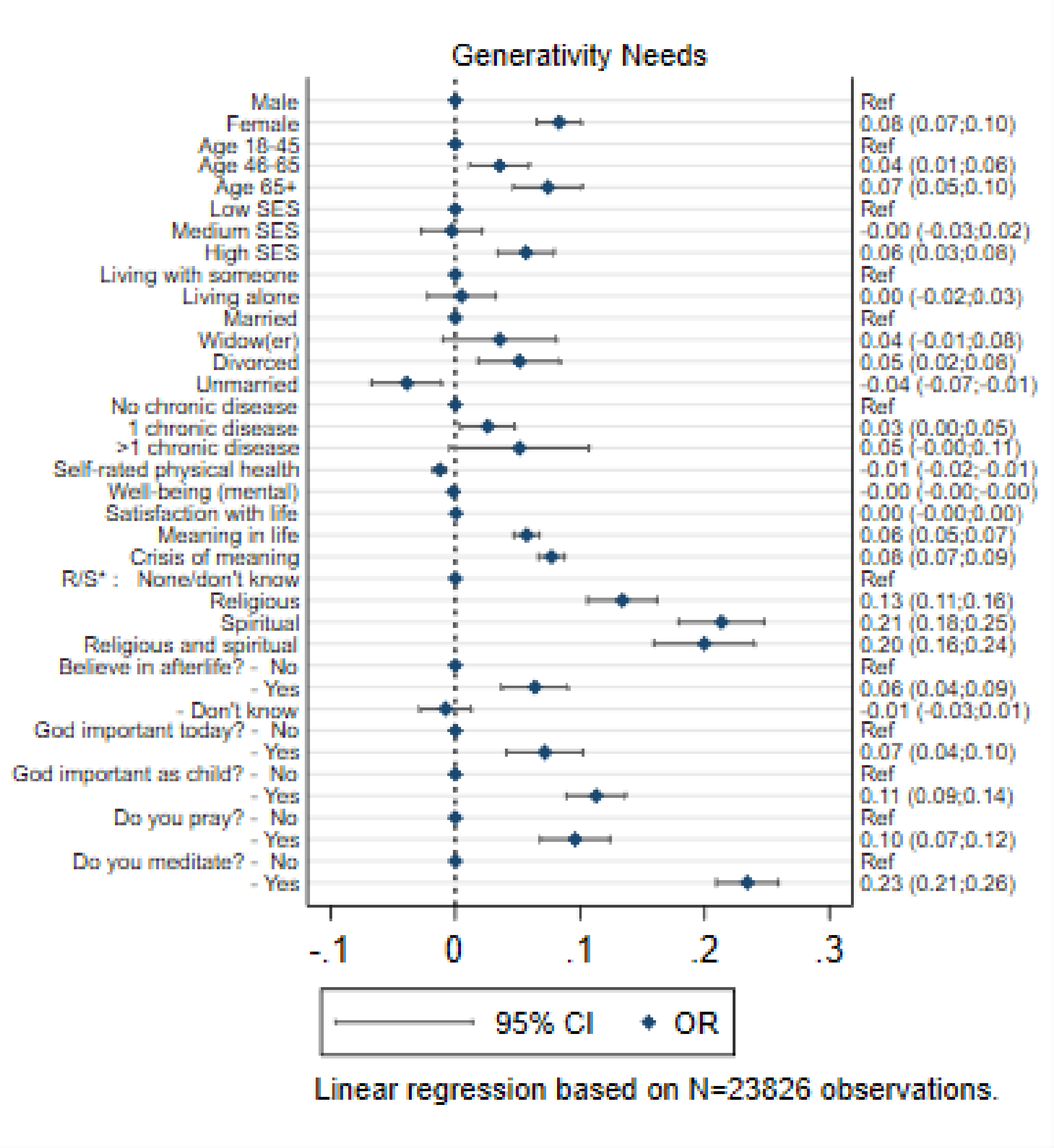

APPENDIX 6.5

FOREST PLOT OF ASSOCIATIONS BY LINEAR REGRESSION OF INNER PEACE NEEDS WITH DEMOGRAPHIC VARIABLES AND SPIRITUAL BELIEFS AND PRACTICES (N=23,826).

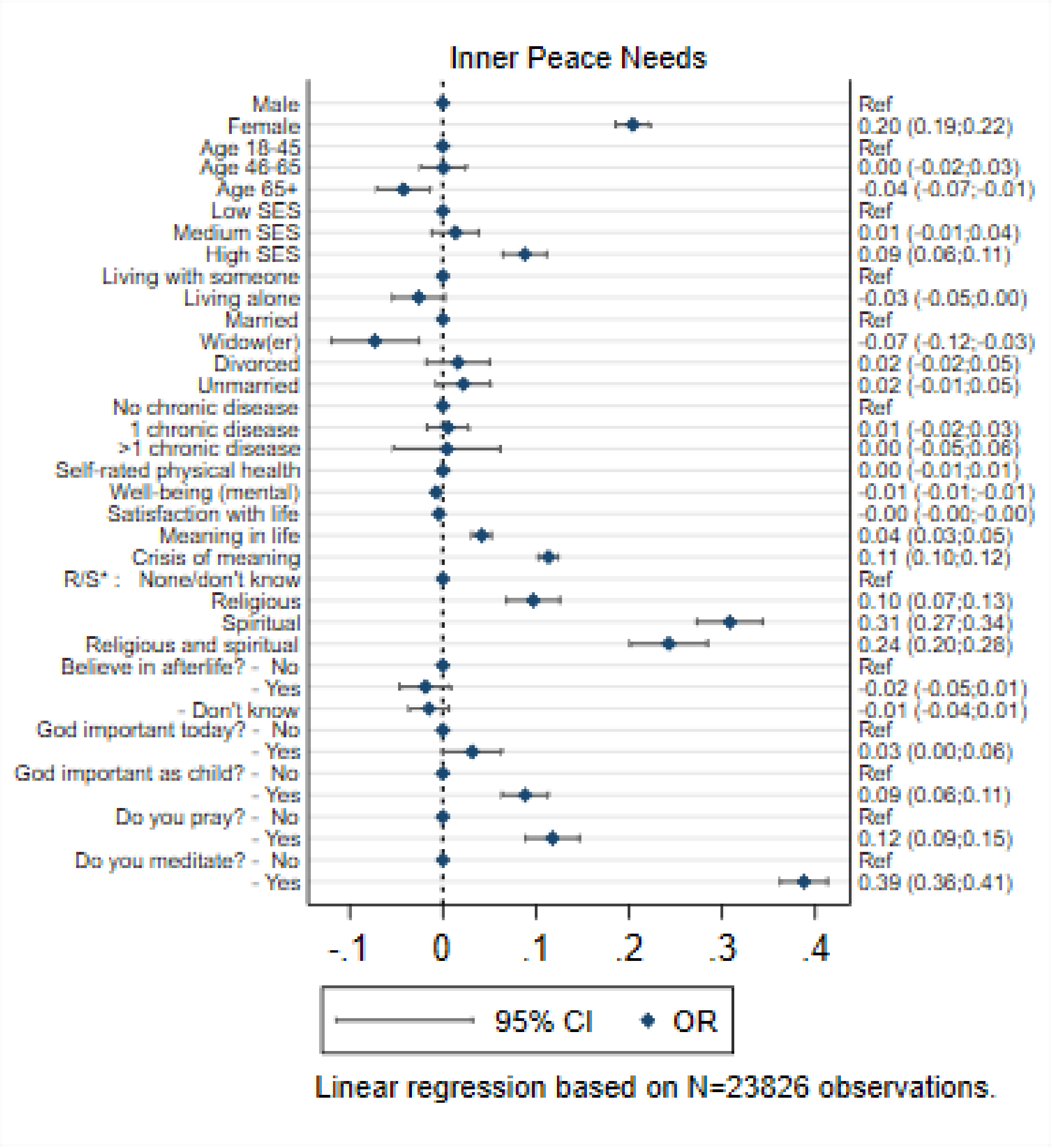

Supplement: Supplementary appendices [file mmc1.pdf]
